# Biological effects of dental resin composites on human gingival fibroblasts: a systematic review of *in vitro* evidence

**DOI:** 10.1007/s00784-026-07149-3

**Published:** 2026-10-05

**Authors:** Elmira Boloori, Dagmar E. Slot, Bruno G. Loos, Teun J. de Vries, Fridus G.A. van der Weijden

**Affiliations:** Department of Periodontology, Academic Centre for Dentistry Amsterdam (ACTA), University of Amsterdam and Vrije Universiteit Amsterdam, Gustav Mahlerlaan 3004, 1081 LA Amsterdam, The Netherlands

**Keywords:** Resin composite, Gingival fibroblasts, Biocompatibility, Cytotoxicity, Viability, Inflammation

## Abstract

**Objective:**

This systematic review summarized and synthesized the *in vitro* biological effects of resin composites (RCs) on human gingival fibroblasts (HGFs) to clarify their isolated cellular biocompatibility.

**Materials and methods:**

A systematic search was conducted in the MEDLINE/PubMed database using a PECOS framework to identify *in vitro* studies evaluating HGF responses to RCs. Two independent reviewers screened literature, extracted data, and assessed methodological quality (QUIN tool) and certainty of the evidence (GRADE framework).

**Results:**

Thirty-eight studies comprising 185 distinct experiments across eight outcome measures were included. Marked heterogeneity was observed regarding RC composition (7 categories), specimen geometry, cell lines, and experimental durations. When evaluating cellular survival and damage independently, 27% of cytotoxicity experiments reported significant cell damage/death, while 45% of cell viability assays demonstrated a decreased metabolic activity. DNA damage was prominent, with 50% of 18 experiments reporting significant alterations. Significant increases occurred in reactive oxygen species production (71%) and inflammatory mediator release (62%). Conversely, 85% of morphology experiments showed no significant alterations. The overall certainty of evidence was graded as low to moderate.

**Conclusion:**

Dental resin composites can induce notable, independent adverse biological responses in human gingival fibroblasts *in vitro*, characterized by oxidative stress, inflammatory mediator release, DNA damage, and compromised viability. While highlighting a clear potential for toxicity, the low-to-moderate certainty and lack of physiological simulations leave their direct clinical significance open to further investigation.

**Clinical relevance:**

Synthesized evidence indicates that dental RCs exert moderate *in vitro *biological effects on gingival fibroblasts, including DNA-damage and inflammatory responses.

**Supplementary information:**

The online version contains supplementary material available at https://doi.org/10.1007/s00784-026-07149-3.

## Introduction

Resin composite (RC) restorations are the restorative materials of choice in modern dentistry, replacing amalgam in numerous clinical settings. This transition is driven by increasing concerns regarding the biocompatibility, toxicity, and environmental impact of mercury-containing amalgams, as well as by heightened patient demand for esthetic, tooth-colored restorations [1]. Modern RCs are the product of decades of advancements, evolving from initial methacrylate monomer-based composites introduced in the 1940s [2] to complex systems featuring inorganic fillers within an organic resin matrix aimed at optimizing esthetic, mechanical, and biological properties [3].

The resin matrix typically comprises dimethacrylate monomers such as bis-GMA, UDMA, and TEGDMA, along with co-monomers, photoinitiators, inhibitors, and stabilizers. These components are supplied in a viscous paste form to facilitate clinical handling and esthetic blending with the natural dentition. Inorganic filler particles, such as silica, ceramic, quartz, or hybrid composites, are incorporated to enhance mechanical resilience, reduce polymerization shrinkage, and improve the surface smoothness [3–6]. This mixture paste is then polymerized into a hard composite material.

Polymerization occurs through light-activation (within 400–490 nm), initiating free radical crosslinking within the resin matrix [7, 8]. However, complete monomer conversion during polymerization is rarely achieved, leading to residual monomers and additives capable of leaching into adjacent periodontal tissues [9]. Literature estimates suggest that between 15 and 50% of these monomers may remain unpolymerized post-curing, depending on factors such as light intensity, exposure time, cavity depth, and composite thickness [10]. Incomplete polymerization allows residual monomers, co-monomers, initiators, stabilizers, and contaminants to leach into surrounding tissue, raising concerns regarding their biocompatibility. The challenge hinges primarily on these leachable organic components, as the inert filler particles are generally considered biocompatible [11]. Leached monomers and additives can affect surrounding periodontal tissues, especially when restorations are placed subgingivally or at the gingival margin, potentially inducing inflammatory responses, or tissue irritation [5, 12–14].

However, determining the exact driver of this localized gingival inflammation presents a distinct clinical challenge.* In vivo* clinical studies are frequently limited by population heterogeneity and long-term follow-up constraints. Furthermore, they generally cannot isolate whether gingival irritation is caused by surface roughness, plaque accumulation, or the direct chemical toxicity of monomer leaching.

Consequently, biocompatibility is most effectively evaluated *in vitro* by isolating the material and measuring specific cellular reactions, with cell viability and cytotoxicity serving as the primary established outcomes [10, 14, 15]. The primary periodontal tissues of concern are the gingival epithelium and the underlying connective tissue fibroblasts, which are directly exposed to potential leachates. Accordingly, most *in vitro* research has focused on fibroblast cultures. Understanding their cellular responses, including viability, proliferation, and inflammatory cytokine production, is essential for evaluating the biocompatibility and predicting potential adverse effects of RC restorations at or below the gingival margin.

## Objectives

This systematic review focuses on the chemical and cellular toxicity of these materials, addressing an important component of their overall clinical safety profile. By systematically summarizing the existing* in vitro* literature, this review aims to synthetize the biological effects of RCs, on human gingival fibroblasts (HGF), thereby providing insights into their periodontal biocompatibility.

Null Hypothesis (H_0_): Resin composite restorations exert no significant biological effects on HGF *in vitro* and do not influence parameters associated with cellular biocompatibility.

## Materials and methods

This systematic review was conducted in accordance with PRISMA 2020 [16] as the primary reporting framework, supplemented by Guidelines of Meta-analysis Of Observational Studies (MOOSE) where applicable. (Supplementary Table S.9.1 and Table S.9.2) [17].

### Focused question: Population, Exposure, Comparison, Outcome, Studies (PECOS)

Based on the following items:

Cell type / Model system (population): gingival fibroblasts

Exposure: dental RCs

Comparator: dentin or enamel or tissue culture material treated plastic

Outcome: cellular endpoints

Study design: *in vitro*

What are the effects of exposure to dental resin composites on cellular outcomes in gingival fibroblasts compared with dentin, enamel, or tissue-culture-treated plastic surfaces in *in vitro* studies?

### Search strategy

The National Library of Medicine in Washington, D.C. (MEDLINE-PubMed) served as the database for an extensive literature search aimed at identifying relevant scholarly articles to meet the study’s objectives. A methodical search strategy was implemented utilizing a framework designed to encompass any published research assessing the impact of dental RCs on fibroblasts. For specific search terms used in the strategy see Table 1.

**Table 1:** Mesh terms used in PubMed

Mesh terms PubMed
((((“fibroblastic“[All Fields] OR “fibroblasts“[MeSH Terms]) OR “fibroblasts“[All Fields]) OR “fibroblast“[All Fields]) OR “fibroblasts“[MeSH Terms]) AND ((((“composite resins“[MeSH Terms] OR ((((((((“composite“[All Fields] OR “composite s“[All Fields]) OR “composited“[All Fields]) OR “composites“[All Fields]) OR “compositing“[All Fields]) OR “composition“[All Fields]) OR “compositional“[All Fields]) OR “compositions“[All Fields]) AND “resins, synthetic“[MeSH Terms])) OR ((((((((“composite“[All Fields] OR “composite s“[All Fields]) OR “composited“[All Fields]) OR “composites“[All Fields]) OR “compositing“[All Fields]) OR “composition“[All Fields]) OR “compositional“[All Fields]) OR “compositions“[All Fields]) AND ((((“resins, synthetic“[MeSH Terms] OR (“resins“[All Fields] AND “synthetic“[All Fields])) OR “synthetic resins“[All Fields]) OR (“dental“[All Fields] AND “resins“[All Fields])) OR “dental resins“[All Fields]))) OR ((“composite resins“[MeSH Terms] OR (“composite“[All Fields] AND “resins“[All Fields])) OR “composite resins“[All Fields])) OR ((((“composite resins“[MeSH Terms] OR (“composite“[All Fields] AND “resins“[All Fields])) OR “composite resins“[All Fields]) OR (“resin“[All Fields] AND “composite“[All Fields])) OR “resin composite“[All Fields]))

### Screening and selection

Titles and abstracts of publications from the search were screened by two reviewers (EB and TJdV) using the Rayyan web application [18] up to February of 2026. The eligibility criteria were:

- *In vitro* studies
- Publication in English
- Human gingival fibroblasts
- Use of dental RC
- Polymerization with blue light
- Evaluating biocompatibility endpoint measure(s)

Exclusion criteria were:

- Other materials than RC
- Other cell-types than human gingival fibroblasts
- Resin materials like glass-ionomer, flowable, cements, adhesives, bulk, 3-D or CAD/CAM

Reviewers independently evaluated the titles and abstracts yielded from the search, classifying them as included, excluded, or undecided. This initial screening was conducted in a blinded manner to ensure unbiased selection. Following this screening, the process was unblinded, and any ‘conflicts’ identified by the Rayyan application were deliberated among the reviewers. Any disagreements between reviewers regarding quality assessment were resolved through further discussion with author DES. The use of independent, blinded screening followed by consensus-based resolution of disagreements was applied to minimize the potential for selection bias. Once a consensus was reached, the full-text articles of the included studies were retrieved for subsequent data extraction.

### Assessment of heterogeneity

A comprehensive examination was conducted on selected studies included in this systematic review. Key heterogeneity factors were identified, and analyzed:

- Composite brand and composition
- Specimen sizes and utilization
- Type of controls
- Type of assays used
- Outcome measures

### Quality assessment

Two reviewers (EB and TJdV) independently assessed the risk of bias of the included studies using the Quality Assessment Tool for *In Vitro* Studies (QUIN tool) [19]. Before initiating the formal evaluation, the reviewers performed a calibration exercise by jointly reviewing the checklist questions to promote a consistent interpretation and application of the assessment criteria. The QUIN tool for risk of bias comprises 12 criteria (Supplementary Table S.6.1), each of which is scored on a three-point scale: adequately specified (score = 2), inadequately specified (score = 1), not specified (score = 0). The QUIN tool was selected as the most recent tool available for assessing the methodological quality and risk of bias of *in vitro* studies. The scores for the individual criteria were summed and converted into a percentage of the maximum possible score. This percentage was used to classify the overall risk of bias as low, medium, or high, according to the predefined QUIN thresholds. Thus, the overall classification reflected the aggregate assessment across all QUIN criteria rather than the result of a single criterion. Any disagreements between reviewers regarding the risk-of-bias assessment were resolved through further discussion and, when necessary, consultation with a third reviewer (DES). Studies were classified as having a low risk of bias if they scored > 70% or higher, a moderate risk of bias with scores between 50% and 70%, and a high risk of bias if the score was lower than 50% [19] .

### Data extraction and analysis

Data from the papers meeting the eligibility criteria were extracted and after extraction analyzed by two reviewers (EB & TJdV). The reviewers conducted their assessments independently and were blinded to each other’s results throughout the process. A descriptive data presentation was employed across all studies to convey findings. The cellular outcomes were categorized into distinct domains to allow separate assessment of different aspects of biocompatibility. The parameters of interest included cytotoxicity, cell viability, proliferation/adhesion, inflammatory markers, DNA-damage, Reactive Oxygen Species (ROS) production, cell morphology, and cell attachment.

### Grading the ‘body of evidence’

The certainty of the evidence was assessed using the GRADE (Grading of Recommendations, Assessment, Development, and Evaluation) framework. Because the evidence synthesized in this review was derived exclusively from *in vitro* studies, GRADE domains were considered in the context of laboratory evidence rather than clinical treatment effects. In particular, risk of bias, inconsistency, indirectness, imprecision, and publication bias were evaluated based on the characteristics and limitations of the included *in vitro* studies. The resulting GRADE ratings were interpreted as reflecting the certainty of the available evidence for the investigated outcomes and should not be interpreted as indicating the certainty of clinical effects or direct clinical applicability. Three reviewers (EB, DES & GAW) assessed the quality of evidence along with the certainty of the evidence.

## Results

### Search and selection results

**Fig. 1:**
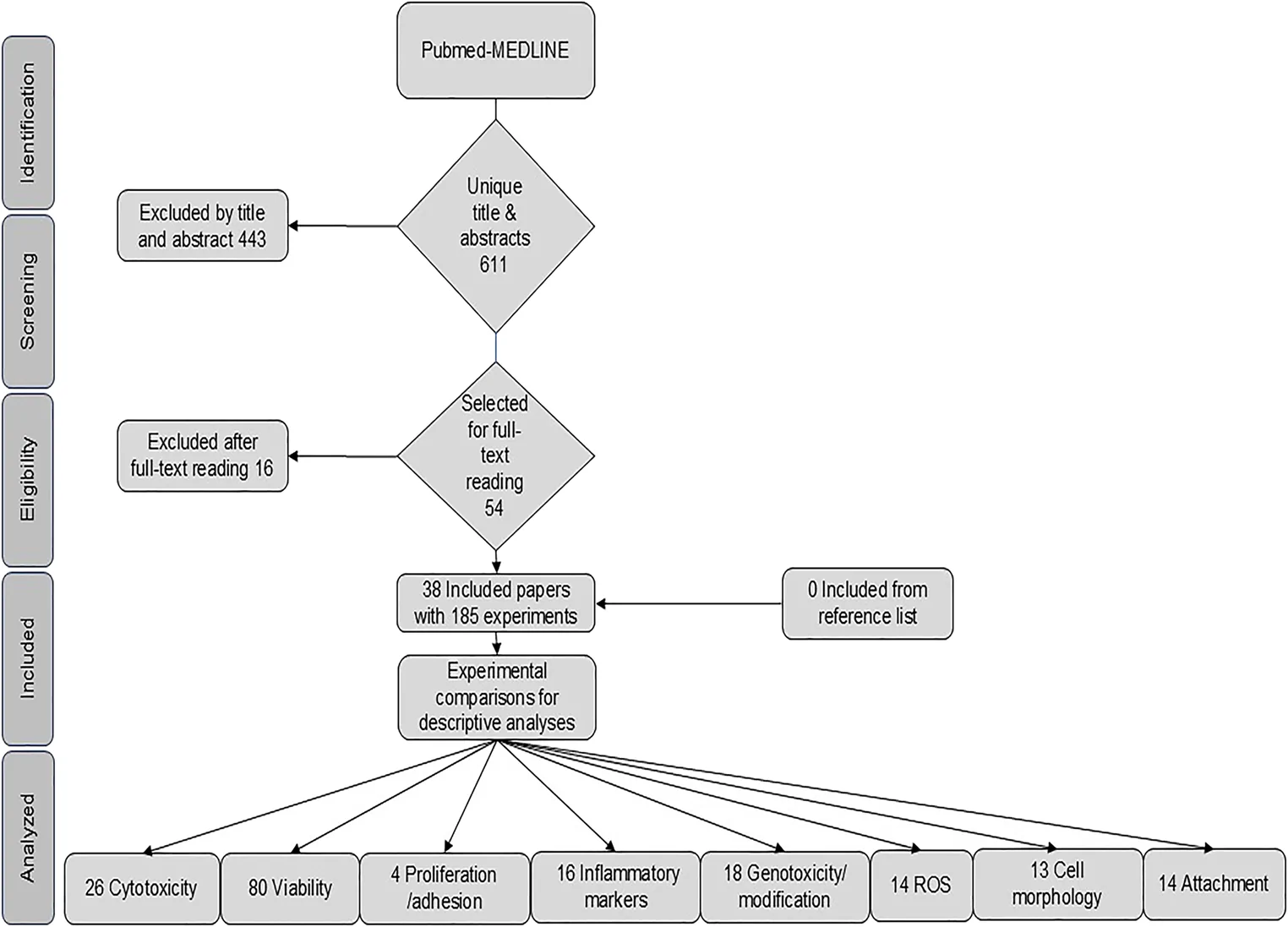
Flow chart for search and selection

The search identified 611 unique papers. Following initial screening of titles and abstracts, 54 articles underwent full-text assessment, of which 38 met the eligibility criteria and were included in the systematic review. (Fig. 1) [6, 20–55]. Sixteen studies were excluded from the analysis based on specific criteria related to the materials utilized. These studies did not exclusively examine RCs, or they did not use HGF as cell model. Additionally, the composites assessed were flowable or indirect composites, which are prefabricated, and/or polymerization was not conducted using blue light. To standardize the diverse materials and outcome measures across the 38 included studies, data was analyzed at the experimental level. Consequently, 185 distinct experiments involving eight different outcome measures were identified and evaluated (Fig. 1).

### Heterogeneity

#### Design

Considerable heterogeneity was found across the included studies in terms of study design, particularly with respect to the research protocols used, including the assays employed, types of RCs, curing times, specimen sizes, and fabrication methods. Supplementary Table S.1 provides an overview of the studies included and their respective characteristics.

#### Resin composites

A total of 52 brands of RCs were used (Supplementary Table S.2). These brands were categorized according to their main content (Supplementary Table S.2) based on the compositions reported in the individual studies or obtained from available online resources detailing the composition of the respective materials. The RCs were subsequently grouped according to their main constituents to facilitate systematic comparison of the findings across studies.

**Table:** 

This RC categorization resulted in seven groups:	
Group A:	BisGMA/BisEMA+TEGDMA
Group B:	UDMA+TEGDMA/Bis-GMA/BisEMA
Group C:	BisGMA+BisphenolA glyceralate dimethacrylate/Bis-GMA, triethylene glycol dimethacrylate Bis-GMA, Hydrophobic-aromatic-dimethacrylate
Group D:	Urethane-dimethacrylate, tricyclodecane-imethanol-dimethacrylate
Group E:	BisGMA+Bisphenol-A-dimethacrylate+Bisphenol-Amethacrylate, TEGDMA, BisEMA
Group F:	ORMOCER^®^
Group G:	Unclassified

Twelve different experiments were performed on composites in Group A, while fifty experiments were conducted on those in Group B. Group C composites were used in fourteen experiments, whereas Group D composites were included in 4 different experiments. Group E composites were examined in 63 experiments whereas in Group F 8 experiments were included. Fourteen brands could not be categorized by content (Group G) and 34 experiments evaluated composites in this group. Table 2 shows the different groups and the number of experiments conducted for each outcome measure.

**Table 2.**
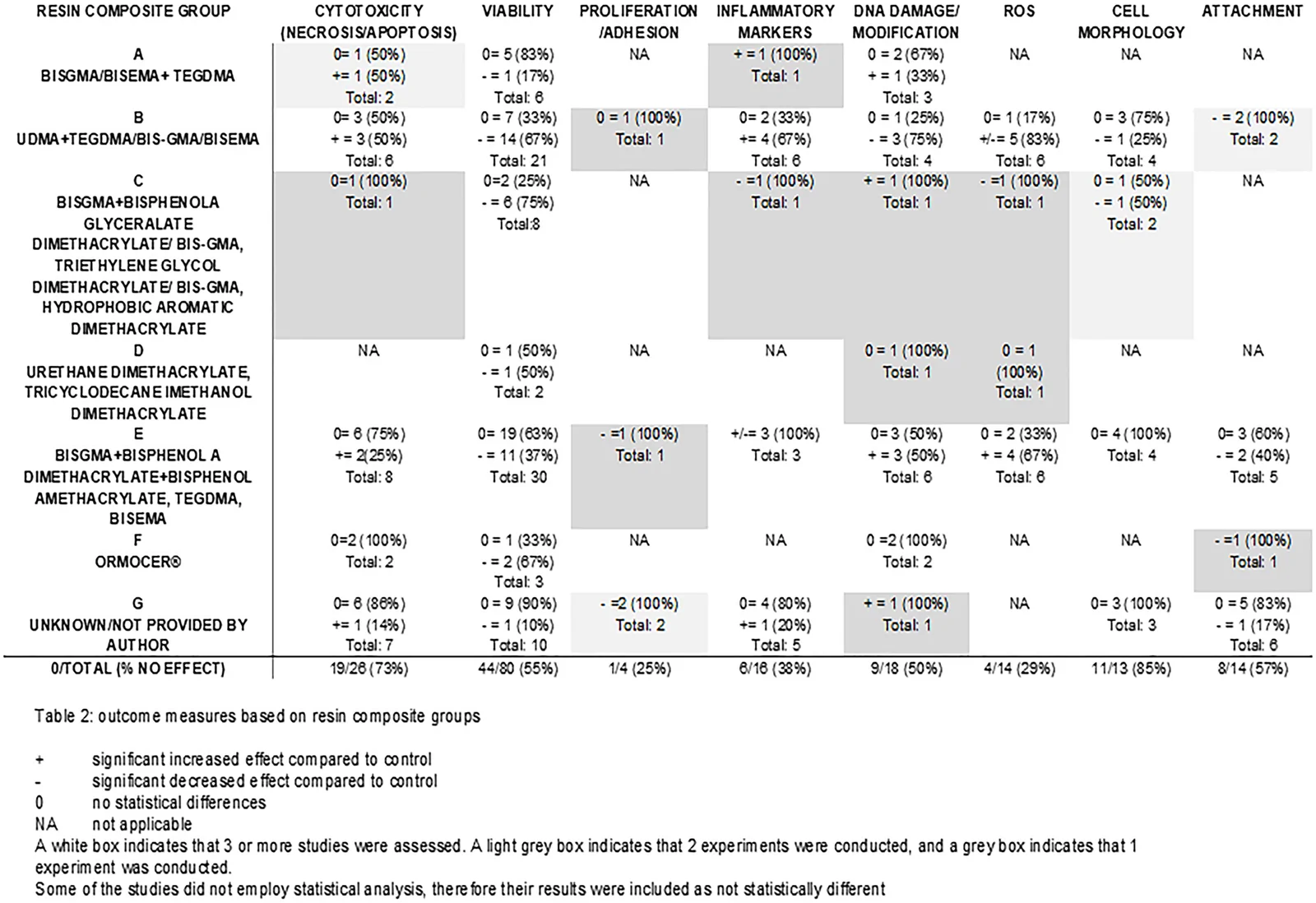
Outcome measures based on resin composite groups

#### Specimen size

Among the 38 included studies, seventeen [23–27, 33, 34, 36, 43, 47–49, 51, 54, 56] used specimens with volumes ranging from 1 to 40 mm³, nine studies [6, 20, 32, 35, 38, 41, 44, 50, 55] used specimens with volumes between 41 and 80 mm³, and seven studies [22, 39, 40, 42, 45, 46, 52] employed specimens with volumes exceeding 80 mm³. Three studies [21, 28, 35] measured specimen size based on weight. Two studies [21, 28] used specimens weighing between 0.1 and 0.2 mg, and one study used specimens weighing 76.6 mg. In four studies [29–31, 53], the specimen sizes were not specified (Supplementary Table S.7).

#### Usage of specimens

The majority of the studies used eluates to conduct their tests, and the leakage times varied across the studies. Seven experiments [20, 21, 25, 26, 28, 41, 44] were performed directly on the specimens (Supplementary Table S.1 and S.10).

#### Controls

Many of the included studies (36) used tissue culture plastic as controls, while two studies employed alternative materials: polyethylene discs and glass (Supplementary Table S.1) [20, 25].

#### Biocompatibility assays

A total of 26 different assays were employed to analyze the biocompatibility of the RCs. The MTT assay was the most frequently used, with 19 experiments employing this test to measure cytotoxicity or viability and 2 experiments using this assay for cell adhesion-measurements. The remaining assays were applied in five or less experiments (Supplementary Table S.5).

#### Outcome

A total of eight different outcome measures were found and grouped to assess the biocompatibility of RCs. Some studies have studied multiple outcome measures. Viability was the most common outcome measure, reported in 80 experiments, followed by cytotoxicity with 26 experiments. Additionally, 13 experiments examined cell morphology and 14 examined cell attachment, 16 investigated inflammatory markers, 18 assessed DNA-damage or genomic modifications, 14 experiments evaluated the effects on ROS production, and 4 experiments measured cell proliferation (Table 2).

### Data analysis

The seven composite groups across eight outcome measures theoretically totaled into 56 combinations. Of these, 16 combinations had no experimental data, 11 were based on a single experiment, and seven were based on two experiments. The remaining 22 outcomes were supported by three or more experiments. Coverage varied by group: Groups B and E were evaluated across all eight outcome measures, Group G across seven, Group C across six, and Groups A and D across four measures each. (Table 2)

### Per composite group

#### Composite group A: “BisGMA+TEGDMA”

For Group A, two experiments investigated cytotoxicity. One reported a significant increase while the other found no statistical difference. Of the six experiments examining cell viability, only one showed a significant decrease compared to the control. A single study on inflammatory markers reported a significant increase, and two out of three experiments regarding DNA-damage showed no significant difference (Table 2).

#### Composite group B: “UDMA+TEGDMA”

For Group B, outcome measures were distributed across all categories. Cytotoxicity results were split between no difference (*n* = 3) and significant increase (*n* = 3). Viability showed the most activity, with 14 of 21 experiments reporting a significant decrease. While a single study on proliferation reported no difference, inflammatory markers significantly increased in four of six cases. DNA-damage was reduced in three of four studies, and ROS production showed varied effects in five of six experiments. Finally, cell morphology mostly showed no change (2 out of 3 experiments), while cell attachment significantly decreased in both conducted experiments. (Table 2).

#### Composite group C: “BisGMA+Bisphenol-A-glycerol-dimethacrylate”

In Group C, research spanned six outcome measures. Cytotoxicity remained unaffected in the single study performed. Viability decreased significantly in 75% of experiments (6 of 8), while inflammatory markers and ROS both showed a significant decrease in their respective single experiments. Conversely, DNA-damage significantly increased in its sole study. Finally, cell morphology results were divided, with one experiment showing no statistical difference and another reporting a significant decrease (Table 2).

#### Composite group D: “BisGMA+Bisphenol-A-dimethacrylate+Bisphenol-A-methacrylate+TEGDMA”

Research for Group D was limited to four outcome measures. While viability results were split between no difference and a significant decrease (*n* = 1 each), the single experiments conducted for inflammatory markers, DNA-damage, and ROS all reported no statistical differences compared to the control (Table 2).

#### Composite group E: “BisEMA+TEGDMA”

For Group E, research covered all eight outcome measures. Cytotoxicity showed no statistical difference in 75% of experiments (6 of 8), while viability decreased significantly in 11 of 30 experiments. Single experiments for proliferation and 3 experiments for inflammatory markers showed significant differences. Both DNA-damage and ROS results were split, with 50% showing no difference and the other half showing significant increases. Finally, cell morphology was consistently unaffected (experiments *n* = 4), and attachment decreased in 40% of the five experiments conducted (Table 2).

#### Composite group F: “ORMOCER^®^”

Research for Group F was restricted to three outcome measures. Both cytotoxicity studies reported no statistical difference. Viability results showed no difference in one study and a significant decrease in two others. Finally, the single experiment conducted for cell attachment reported a significant decrease compared to the control (Table 2).

#### Composite group G: “Unclassified”

For Group G, research covered seven outcome measures. Cytotoxicity showed no statistical difference in 86% of experiments (6 of 7). Viability remained largely unaffected, with 90% of experiments (9 of 10) reporting no difference. Proliferation significantly decreased in both conducted experiments, while inflammatory markers mostly showed no change (4 of 5). Single experiments for DNA-damage and ROS reported a significant increase and no statistical difference, respectively. Lastly, cell attachment showed no statistical difference in five of six experiments (Table 2).

### Overall results

#### Per outcome measure

Across all seven composite groups, the cumulative analysis of the eight outcome measures revealed that ROS production was the most frequently affected parameter, with no statistical differences reported in only 29% of 14 experiments. Proliferation/adhesion also showed consistency, as only 25% of the four conducted experiments reported no effect. Cell morphology remained the most stable outcome measure, with 85% of 13 experiments showing no statistical difference. Cytotoxicity was largely unaffected in 73% of 26 cases. Viability, the most extensively researched measure, in a total of 80 experiments, showed no statistical difference in 55% of these experiments. Cell attachment and DNA-damage/modification remained unaffected in 57% and 50% of their respective experiments. Finally, inflammatory markers showed no statistical difference in 38% of the 16 experiments performed (Table 2). For an extensive analysis of the results, see Supplementary Table S.8.

### Quality assessment

#### Risk of bias

Supplementary Table S.3 and Table S.6.2 present the quality assessments of the included studies. Of the 38 studies, 37 exhibited a medium risk of bias, while 1 [53] demonstrated a low risk of bias.

### Grading the body of evidence

Due to substantial methodological heterogeneity among the included studies, their characteristics could not be meaningfully summarized in a single concise table and are therefore presented in detail in supplementary Table S.10. These characteristics informed the methodological quality assessment (Supplementary Table S.4) and the assessment of the certainty of the evidence according to the GRADE framework [57]. While the risk of bias was assessed as medium across the included studies, the consistency of findings for most outcome measures was, at best, fair, indicating heterogeneity in study results. With moderate certainty, evidence suggests that RC exhibits some influence on cytotoxicity and cell viability, although the magnitude of these effects appear limited. Furthermore, moderate-certainty evidence indicates that RCs impact inflammatory responses, whereas no discernable effect on cellular morphology was observed. For other outcome measures, the certainty of the evidence ranged from low to very low, precluding robust conclusions. Proliferation assessment was particularly limited, with insufficient data to permit any meaningful evaluation (Table 3).

**Table 3:** Summary of grading the certainty of the evidence

Determinants of the Quality	Cytotoxicity (necrosis/apoptosis)	Viability	Proliferation /Adhesion	Inflammatory markers	DNA-damage/ modification	ROS	Cell morphology	Attachment
Strength of the evidence for the observation based on the quality and body of evidence	Moderate	Moderate	Very low	Low	Very low	Moderate	Moderate	Low
Rating of the certainty of the evidence	With moderate certainty, there appear to be some effects on cytotoxicity; however, the use of various types of composites is not discouraged based on this outcome measure. Considering the fair consistency of results and the fact that 73% of the experiments show no significant effect of resin composites on gingival fibroblast cells, the evidence suggests that these materials may generally pose a low risk for impacting gingival fibroblast cell health adversely.	With moderate certainty, there appear to be some effect on viability; however, the use of various types of composites is not discouraged based on this outcome measure. Considering the inconsistency of results and the fact that 55% of the experiments show no significant effect of resin composites on gingival fibroblast cells, it suggests that these materials may pose some risk for viability of gingival fibroblast.	There is insufficient data to rate the evidence	With low certainty, resin composites appear to have a negative effect on inflammation. Considering the poor consistency of results, it is possible that these materials may generally pose a risk for impacting gingival fibroblast cell health adversely and it is advisable to be mindful of this effect.	With very low certainty, there is some effect of resin composites on gene toxicity/ modification; however, the use of these materials is not discouraged, as there is a lack of evidence that resin composite has a negative effect on DNA of gingival fibroblast.	With moderate certainty, in line with inflammatory markers, resin composites appear to lead to increased reactive oxygen species (ROS) production, and this aspect should be considered.	With moderate certainty, resin composites do not appear to have any effect on cell morphology.	With low certainty, the attachment of gingival fibroblasts to resin composites appears to be impaired, which may lead to deepening of periodontal pockets in susceptible patients. Therefore, this unfavorable aspect needs to be heeded.
Overall rating of the evidence and recommendation	There is considerable contradiction among the reviewed studies, therefore, the certainty of the evidence ranges from very low to moderate. The use of resin composites is not discouraged, as no significant differences have been observed among various types. However, given that resin composites may induce some degree of inflammation, special attention should be given to patients susceptible to gingivitis and periodontitis. Furthermore, there is a critical need for standardization in assessing biocompatibility, ensuring that outcome measures are comprehensive and that consistent protocols and assays are employed.							

Conclusions based on findings on body of the estimated evidence profile [57] and appraisal of certainty and the strength of the evidence of the observation regarding the effect of various resin composites on gingival fibroblast cells on the parameters of interest. Supplementary Table S4 provides a complete overview of the details of the GRADE assessment

## Discussion

This systematic review aimed to evaluate the biological effects of dental RCs on human gingival fibroblasts. Given the increasing clinical use of these materials and the importance of understanding their implications for periodontal health, it is relevant to evaluate their safe use in terms of their effects on periodontal cells such as gingival fibroblasts. Synthesis of the available scientific evidence showed that across composite resin groups, biological responses varied depending on monomer composition. UDMA+TEGDMA (Group B) and BisEMA-containing materials (Group E) showed consistent reductions in cell viability and increased inflammatory responses. In contrast, BisGMA+TEGDMA (Group A) and unclassified composites of uncertain identity (Group G) generally showed limited or no consistent cytotoxic or inflammatory effects, with most outcomes not differing from controls. Overall, ROS production, cell proliferation, and adhesion consistently exhibited negative effects, whereas cell morphology and cytotoxicity showed fewer significant changes. Taken together, the emerging evidence indicates that the biological effects of composites are formulation-dependent, with no uniform cellular effects on gingival fibroblasts across all materials. Overall, this systematic review suggests that dental RCs exert moderate biological effects on gingival fibroblasts, which may potentially have a negative effect on periodontal health.

### Cellular effects of resin composites

While certain monomer combinations may elicit measurable cellular responses, these effects are not consistently pronounced across formulations or outcome measures. Notably, only 27% of the distinct experiments in the included studies reported a significant cytotoxic effect, whereas roughly 45% demonstrated measurable changes in cell viability. This observation is of interest, considering that viability assessments are commonly employed as primary indicators of cytotoxicity. Conversely, DNA damage was more prominently affected, with 50% of the 18 experiments showing significant DNA damage. These findings suggest that, although immediate viability loss may be limited, RCs may induce genetic alterations which may potentially have long-term consequences [54, 56]. This concern deserves further investigation.

It is important to contextualize these results with the transient nature of leachable components. The vast majority (approximately 85–100%) of total leachable monomers are typically released within the first 24 h after polymerization [58]. However, incomplete polymerization resulting from insufficient curing time or lower light intensity significantly increases the leaching of residual monomers from dental RCs [59, 60]. Most resin monomers and additives that leak following polymerization are furthermore washed away by saliva over the short term [61], reducing the durability of any adverse effects. Nevertheless, monomer release can continue at lower levels for significantly longer periods. Studies have detected ongoing leaching from 7 days to 3 months [62]. Long-term wear and degradation could lead to persistent low-level leaching, which might cumulatively affect periodontal tissues [63]. The relative impact of short-term versus long-term leakage remains a research gap, especially considering that the quantity of residual monomers decreases significantly within one week after initial placement [58].

### Cell morphology and cell attachment

While morphological assessments reveal no significant alterations in cell phenotype or structural integrity, approximately 43% of the experiments in the reviewed studies reported impaired adhesion of gingival fibroblasts to composite surfaces. Particularly, gingival epithelial cells, which constitute the outermost layer of periodontal tissues, remain underrepresented in adhesion research despite their role as the first point of contact at the tooth-gingiva interface. The integrity of this epithelial seal is important; cell attachment to restorative materials facilitates soft tissue integration, thereby protecting the subgingival environment from microbial infiltration and subsequent inflammatory cascades [64]. Current evidence suggests that this biological integration is temporally sensitive. Specifically, epithelial cell attachment is reduced immediately following polymerization, likely due to the high initial efflux of unreacted (co)monomers, whereas cell attachment levels normalize after a one-week leaching period [65]. Consequently, the transient cytotoxicity associated with early-stage leaching may pose a window of vulnerability, potentially compromising long-term biological performance and periodontal stability by creating an environment conducive to colonization by periodontal pathogens. Since the epithelium is the first tissue interface with restorative materials, including epithelial responses in future research may improve the clinical relevance of* in vitro* models.

### Inflammatory and oxidative responses

A considerable number of the reviewed experiments documented that exposure to RCs upregulates pro-inflammatory mediators (62%; 10 out of 16 experiments) and heightened reactive oxygen species (ROS) generation (71%; 10 out of 14 experiments) in gingival fibroblasts. These findings indicate that the initial elution of unreacted monomers triggers pro-inflammatory pathways and oxidative stress. In a clinical setting, this cellular stress likely manifests as localized gingival irritation or gingivitis, particularly during the acute phase following restoration placement when monomer release is at its peak.

While a recent systematic review on deep margin elevation (DME) reported no remarkable differences in probing pocket depth or plaque index [66], these parameters may not fully capture the early biological response. Therefore, reliance on traditional periodontal measurements may mask early-stage iatrogenic effects. Focused clinical longitudinal studies are required to determine whether the transient biologic effects observed *in vitro* translate into permanent attachment loss or chronic periodontal pathology around resin-composite-restored teeth.

### Limitations of current literature

#### *In vitro* studies

As noted, biocompatibility data regarding RCs predominantly derive from *in vitro* models, whereas *in vivo* investigations remain scarce. Although* in vitro* designs offer greater control over experimental conditions, they cannot fully replicate the complex clinical environment. Consequently, several factors that may influence the performance of restorations in clinical settings cannot be adequately represented in laboratory models.

In clinical practice, multiple variables can influence impact RC polymerization. For instance, restricted access to the prepared cavity can impede optimal positioning of the curing unit, resulting in incomplete polymerization and increased leaching of RC components [67]. Additionally, RC placement requires adequate moisture control to achieve optimal polymerization [68]. Maintaining adequate isolation is frequently challenged by salivary contamination and bleeding during subgingival restorations. Finally, the continuous clearance and dilution of leached substances by saliva may reduce their persistence and concentration in the oral cavity, raising questions regarding the extent to which the adverse effects observed *in vitro* persist under clinical conditions. Consequently, caution is warranted when extrapolating *in vitro* biocompatibility data directly to actual clinical scenarios.

#### Short-term focus

Most included studies evaluate short-term responses, typically within hours to one week, which limits the understanding of chronic or cumulative effects. The limited ability of *in vitro* systems to replicate *in vivo* conditions supports the use of longer-term *in vivo* studies to investigate chronic biological responses, such as tissue reactions or immune responses of the gingiva to RCs that could be assessed for instance in the crevicular fluid. Such studies could provide a more comprehensive assessment of the biological effects of RCs on gingival fibroblasts (HGFs) and the gingival tissue, thereby improving the clinical relevance of the evidence.

#### Underreporting of adverse effects

Biocompatibility was evaluated as a secondary parameter rather than the primary study objective in some included studies, often limited to basic viability or cytotoxicity assays. This narrow methodological scope may result in an incomplete profile of the materials’ biological impact, as more complex interactions, such as long-term inflammatory changes, were not consistently assessed. Furthermore, while most studies did not disclose specific funding sources, the inherent focus on material performance may place greater weight on physical properties than on comprehensive biological risk assessments. Consequently, a comprehensive assessment of the biological effects of RCs remains challenging.

#### Complexity of material composition

RCs are complex mixtures of monomers, co-monomers, fillers, and additives, each with distinct leaching potentials [9]. The combined biological effects of multiple components, which are potentially synergistic, are challenging to assess, especially since many studies evaluate single brands or specific ingredients. In the present systematic review, different brands of RCs were categorized into distinct groups based on their main resin ingredient to facilitate comparison. However, for several brands, it was difficult to ascertain the exact composition of the specific RCs evaluated. Material composition can play a role in how gingival fibroblasts respond to resin composites. Because distinct composite formulations release varying amounts and types of unreacted monomers [9], their biological effects can differ. Consequently, cellular responses in gingival fibroblasts may range from mild, transient cellular stress to moderate reductions in cell viability. Nonetheless, across various groups, no apparent differences emerged regarding fibroblast responses, suggesting a baseline level of biological similarity.

#### Heterogeneity in methodologies

A significant limitation across the reviewed studies is the lack of standardized experimental protocols, which hinders direct comparison of findings and the development of conclusive interpretations (Supplementary Table S.10). Also, large variations in specimen preparation are evident. Specimen sizes range from spherical specimens with a 2 mm diameter [30] to large blocks measuring 5 × 5 × 5 mm [45] or discs with a 19 mm diameter [22]. Furthermore, surface treatments and sterilization methods show no consensus. While some researchers used freshly made specimens [20], others employed different sterilization protocols, including 75% ethanol for 60 s [32, 38], UV radiation for 24 h [21], or dry-heat sterilization at 170 °C for one hour [33] (Supplementary Table S.10). Variations in specimen preparation and processing limit cross-study comparability and may influence observed biological responses due to extended cellular exposure to higher concentrations of components.

Curing parameters also contribute to inconsistent outcomes, with polymerization durations spanning from a brief 15 s [20] to 60 s, or varying between 20 and 40 s depending on the specific material [22]. Additionally, the types of assays employed vary considerably. While most studies rely on the MTT assay to measure cellular metabolic activity [23, 24, 44], others utilize diverse indicators such as RNA and protein synthesis inhibition [20], DNA damage via comet assays [28] or real-time cell impedance analysis [38]. This methodological heterogeneity poses a challenge for interpretation. Specifically, the MTT assay reflects mitochondrial dehydrogenase activity rather than direct cell death. A decrease in signal may be erroneously interpreted as high cytotoxicity when it may actually represent a transient metabolic slowdown in viable cells. Consequently, a reliance on metabolic markers can lead to an underestimation of viability or, conversely, a mischaracterization of the true cytotoxic profile. Differences in curing conditions and assay principles may therefore contribute to variability in the observed biological responses and should be considered when comparing cytotoxicity findings across studies.

Furthermore, the criteria for defining biocompatibility differ among studies. According to ISO 10993-5:2009, a material is considered non-cytotoxic if cell viability remains above 70% (i.e., less than 30% reduction) [69]. While this standard provides a threshold, it primarily assesses short-term viability and does not capture other critical biological responses such as genetic stability, inflammatory cytokine production, or effects on cell attachment and migration. Relying solely on viability metrics offers an incomplete picture, as biomaterials can induce subtle biological effects, like DNA-damage or inflammation, that may be more relevant to long-term tissue health.

To enhance comparability and clinical relevance, future research should preferably adhere to standardized testing protocols, encompassing specimen preparation, curing conditions, assay selection, and leakage assessment. A multi-parametric approach, evaluating viability alongside genetic, inflammatory, and attachment responses, would provide a more comprehensive understanding of biocompatibility and better inform clinical choices regarding dental RCs use. Together, these approaches would enable a more comprehensive assessment of the biocompatibility of RCs.

It is also noteworthy that in almost all studies, the controls consisted of tissue culture plastic. A more clinically relevant comparison may be achieved by assessing differences between the biomaterial and naturally occurring dental tissues, such as enamel or dentin, as cellular behavior may differ depending on the substrate, with respect to cell attachment. Ultimately, the use of biologically relevant substrates may improve the clinical relevance of *in vitro* models by more closely reflecting the conditions encountered *in vivo*.

### Limitations

This systematic review was not registered in an online public registry such as PROSPERO prior to commencement, which represents a methodological limitation regarding prospective transparency. Nevertheless, the review was conducted and reported in accordance with established methodological and reporting standards, including the PRISMA [16] and MOOSE [17] statements.

### *In vivo* relevance

While RCs are already in widespread clinical use, their specific effects on periodontal tissues remain poorly defined. Current *in vitro* models often fail to account for complex biological interactions, such as immune responses, and mechanical forces, that modulate leaching and cellular responses *in vivo*.

A key shortcoming in existing research is the lack of continuous saliva flow simulation, which likely facilitates the rapid clearance of the initial, high concentration “burst” of leachables from the oral environment. Consequently, laboratory-tested concentrations of leached RC ingredients may be artificially high relative to the trace amounts remaining in the sulcus after salivary dilution.

Future research should aim to include experiments designed to identify these actual trace concentrations. These clinically relevant thresholds should then be incorporated into* in vitro* models to move beyond basic cytotoxicity toward a more accurate evaluation of how RCs influence periodontal stability and localized gingival health over time.

### Recommendations for future research

To facilitate cross-study comparisons, standardized protocols must be established for specimen dimensions, curing parameters, and leak assessment. Comprehensive biocompatibility profiles should utilize diverse assays, including genetic stability, inflammatory cytokines, and cell attachment, moving beyond simple viability metrics. Furthermore, research should expand from current fibroblast models to include epithelial responses and long-term effects. Finally, independent multicenter longitudinal studies are essential to mitigate bias and ensure clinical relevance.

Overall, considerable heterogeneity was observed across the reviewed studies, resulting in a range of certainty of the evidence from very weak to moderate. Therefore, the current evidence does not warrant discouraging or encouraging the clinical use of RCs and no apparent differences were identified among various composite types. It remains imperative to establish standardized biocompatibility assessments, including comprehensive outcome measures and validated protocols and assays.

## Conclusion

Based on the synthesized evidence demonstrating biological responses, including alterations in oxidative stress, cell viability, inflammatory mediators, and DNA-related endpoints, across multiple resin composites, the available evidence indicates that resin composites can induce measurable biological effects in human gingival fibroblasts under *in vitro* conditions. However, given the methodological heterogeneity, medium risk of bias, predominance of short-term exposure periods, and limited physiological simulation of the *in vitro* models, the clinical significance of these findings remains uncertain.

### Implications for clinical practice

Synthesized evidence indicates that dental RCs may induce measurable *in vitro* biological responses in gingival fibroblasts, including oxidative stress, altered cell viability, and inflammatory and DNA-related responses. However, these findings should not be interpreted as evidence of clinically relevant adverse effects, nor as a basis for discouraging or endorsing the clinical use of resin composites. The clinical relevance of the observed biological responses remains uncertain, particularly because of the predominance of short-term studies, methodological variability, medium risk of bias, and the limited physiological simulation inherent to *in vitro* models. Clinicians should therefore interpret these findings with appropriate caution, while recognizing that the available evidence does not establish the magnitude or clinical relevance of potential adverse effects.

### Implications for research

Standardized testing protocols and multi-parametric biological evaluations are essential for clinically relevant assessments. Future research should incorporate longer exposure periods, standardized experimental protocols, and models that better reflect the complexity of the oral environment. Well-designed clinical and translational studies are needed to determine whether the biological responses observed *in vitro* translate into clinically relevant effects on periodontal and oral tissues.

## Supplementary information

Below is the link to the electronic supplementary material.

Supplementary File 1 (ZIP 658 KB)

## Data Availability

No datasets were generated or analysed during the current study.

## Abbreviations

RCs: Resin Composites
ROS: Reactive Oxygen Species
GRADE: Grading of Recommendations, Assessment, Development, and Evaluation
PECOS: Population, Exposure, Comparison, Outcome, Studies
MTT: 3-(4,5-dimethylthiazol-2-yl)-2,5-diphenyltetrazolium bromide

## Declarations

**Declaration of generative AI in scientific writing**

This manuscript used NLP (Gemini) for proofreading an entirely human-generated text, but no other use of Al was made, the authors reviewed and edited the content as needed and take full responsibility for the content of the published article.
