# Supplementary material for: Biological effects of dental resin composites on human gingival fibroblasts: a systematic review of in vitro evidence
Source: Clin Oral Investig. 2026 Oct 5;30(10):510. doi: 10.1007/s00784-026-07149-3 (PMC13638730; doi:10.1007/s00784-026-07149-3)
Supplement: Supplementary file 1 [file 784_2026_7149_MOESM1_ESM.zip › Table S.1.docx]

**Biological Effects of Dental Resin Composites on Human Gingival Fibroblasts: A Systematic Review of *In Vitro* Evidence**

**Elmira Boloori ^1^** (<https://orcid.org/0000-0002-9048-7988>)

**Dagmar E. Slot ^1^** (<https://orcid.org/0000-0001-7234-0037>)

**Bruno G. Loos ^1^** (https://orcid.org/[0000-0002-8794-552X](https://orcid.org/0000-0002-8794-552X))

**Teun J. de Vries ^1^** (<https://orcid.org/0000-0001-6464-5172>)

**G.A. van der Weijden ^1^** (<https://orcid.org/0000-0002-5075-8384>)

**^1^** Department of Periodontology, Academic Centre for Dentistry Amsterdam (ACTA), University of Amsterdam and Vrije Universiteit Amsterdam, Amsterdam, The Netherlands

**Corresponding author:**

E. Boloori,

Academic Centre for Dentistry Amsterdam (ACTA),

University of Amsterdam and Vrije Universiteit Amsterdam

Department of Periodontology

Gustav Mahlerlaan 3004, 1081 LA Amsterdam, The Netherlands

Phone: +(31)20 5980 179

E-mail: e.boloori@acta.nl

Journal: Clinical Oral Investigations

| **Study #**  **Authors**  **Risk of bias (ROB) (online Supplementary)** | **Study design, duration/detail of experiment**  **Funding** | **Cell culture details** | **Included composite groups (online supplementary)** | **Study procedures:**  **Regimen,**  **Outcome measures** | **Author’s conclusions** |
| --- | --- | --- | --- | --- | --- |
| **1. Caughman et al. 1991**  **ROB: medium** | - Specimen discs - Diameter 10 mm, 1 mm height  - Polymerization for 15, 30, 60 s  - Control: polyethylene discs  - Freshly made specimens used | - HGF  - Primary cells | - 3 different brands  - Groups:  - Unknown or not provided by the authors (F) | - Cytotoxicity by own developed assay (RNA synthesis and Protein synthesis)  - Incubation time 24h and refreshed and incubated for another 24 h  - Freshly made specimens used | For each composite, as the percentage of monomer conversion increased, cellular toxicity decreased. In comparing different composites having similar monomer conversions, it was found that the filler/resin ratio was not the only factor determining the composite's relative toxicity. |
| **2. Willershausen et al. 1999**  **ROB: medium** | - Specimens 0.1 and 0.12 mg/well  - Disinfection with UV light for 24 hours  - Control: Tissue culture plastic | - HGF  - Primary cells from 3 donors | - 2 different brands  - Groups:  - UDMA and TEGDMA (B)  - Unknown or not provided by the authors (G) | - Viability and numbers with tryptan blue exclusion test  - Protein content and Lactic acid with unknown test kits  PGE release with immunoassay | There was a reduction of cell functions, related to the different resin-based materials. Protein content of the fibroblasts was significantly reduced depending on the material tested. Solitaire induced the highest levels of irritation and the greatest reduction in protein content. Lactate production of fibroblasts also showed some significant reduction in contact with particular materials There were only small differences among the tested materials as concerns measured PGE subset2 release of the fibroblasts. |
| **3. Stanislawski et al. 2000**  **ROB: medium** | - Specimen discs diameter 19 mm, 1 mm height  - Polymerization varied from 20-40 s depending on material  - Eluates used  - Control: Tissue culture plastic | - HGF  - Primary cells (unknown n donors) | - 2 different brands  - Group:  - UDMA+TEGDMA/Bis-GMA/BisEMA (B)  - Unknown or not provided by the authors (G) | - Viability on pulp cells MTT  Cellular GSH (glutathione) via mBCI technique (fluorescence) | With the exception of Z100 MP, all the biomaterials induced a depletion of cellular glutathione (GSH) that was variable depending upon the biomaterial eluates. The strongest GSH depletion was with F-2000, Fuji II, and Photac-fil. GSH depletion, with Compoglass and F-2000, was rapid-detectable after one h of cell treatment and complete within 3 h-whereas a longer period of incubation was required for the other biomaterials. Interestingly, the drug cytotoxic effects induced by all the biomaterials were prevented by cell treatment with the antioxidant N-acetylcysteine (NAC). |
| **4. Huang et al. 2002**  **ROB: medium** | Specimen discs  2x3.5x35 mm  - Eluates used  - Control: Tissue culture plastic | - HGF  - Primary cells (unknown n donors) | - 1 brand  - Group:  - BisGMA, Bisphenol A dimethacrylate, Bisphenol A methacrylate, and TEGDMA (E) | - Cytotoxicity measured with MTT assay  - Cell attachment via cell count MTT assay  Incubation time 2 h  - Proliferation with MTT assay incubation time 3 days | The results showed that resin-modified, glass-ionomer cement Fuji II LC, compomer Compoglass, and resin SpectrumTPH (TPH) were cytotoxic to primary human gingival fibroblast cultures by inhibiting cell growth and proliferation. TPH alone had an effect on cell attachment. It was found that TPH was the most cytotoxic repair material among those tested in all cultures. The toxicity decreased in the order of TPH>FLC>CG. |
| **5. Sigusch et al. 2007**  **ROB: medium** | - Specimen discs diameter 4 mm, 2 mm height  - A2 color  3 LCU  - Eluates used  - Control: Tissue culture plastic | - HGF  - Primary cells (1 donor) | - 4 different brands  - Groups:  - BisGMA and TEGDMA (A)  - BisGMA+Bisphenol A dimethacrylate+Bisphenol Amethacrylate, TEGDMA,  BisEMA (E)  - Unknown or not provided by the authors (G) | - Cytotoxicity with MTT assay  - Leakage time 28 days | After 1 day of storage suboptimal results could be observed for the SML/Durafill and optimal results for SML/Grandio combination (approximately 100% cell viability). In addition, the composite Solitaire the SML yielded significantly better results than the other LCUs. Furthermore, the combination of the SML with Z 250 composite showed, after the first day and up to day 28, statistically significantly higher cell viability rates than the combination with the LED or Heliolux II. |
| **6. Franz et al. 2007**  **ROB: medium** | - Specimen discs diameter 4 mm, 2 mm height  - Either fresh or pre- incubation: 1, 2, 7 days, 6 weeks  - Control: glass | - HGF (unknown n donors)  primary  - L929 mouse fibroblasts | - 1 brand  - Group:  - BisGMA and TEGDMA (A) | - Flow cytometry  - Incubation time 72 h  - HGF was used only in 1 experiment  - Cell number was assessed by FCM $\neq$ cytotoxicity | Different ratios of specimen sizes to cell culture parameters (cell layer surface, volume of cell culture medium) produced different results. Three out of four differently designed specimens showed the same behavior in cell culture. |
| **7. Deb et al. 2011**  **ROB: medium** | - Specimen discs 2 mm in diameter, 1 mm height  - Control: Tissue culture plastic | - HF cell line (origin unclear) | - 3 brands  - Groups:  - BisGMA and TEGDMA (A)  - BisGMA+Bisphenol A dimethacrylate+Bisphenol Amethacrylate, TEGDMA,  BisEMA (E) | - Cell proliferation/cytotoxicity with Alamar Blue and MTT assay  - Not well described | Microleakage studies showed that pre-warming had no significant bearing on the results and alamarBlue(®) results showed that the pre-heating did not have an effect on the cytotoxicity however the levels of cytotoxicity varied between the composites that can be attributed to the composition. |
| **8. Sigusch et al. 2012**  **ROB: medium** | - Specimen discs 4 mm in diameter, 2 mm in height  - A2 and C2 color  - 2 light curing units used  - Half cured, half not cured  - Extracts collected at 1, 2, 3, 4, 5, 6, 7, 14, 21, 28 days  - Eluates used  - Control: Tissue culture plastic | - HGF  - Primary cells (1 donor)  - Cell passages of 6-8^th^ used | - 3 brands  - Groups:  - BisGMA+Bisphenol A dimethacrylate+Bisphenol Amethacrylate, TEGDMA,  BisEMA (E)  - Unknown or not provided by the authors (G) | - Cytotoxicity measured by MTT assay  - Incubation time of 48 hours | With the non-polymerized samples, essentially no influence of the composite shades investigated on HGF viability was detected, with the exception of the Solitaire material, where a higher cytotoxicity of the shade C2 in the non-polymerized state was found at the end of the observation period. After polymerization of the different composites, the cytotoxic reaction observed for the extracts of shade C2 was stronger than that observed for A2. After polymerization with the Heliolux II (HLX) LCU, the extracts of composites Grandio and Solitaire C2 were significantly more toxic than those of the A2 shade. The extracts of the Grandio composite showed the least cytotoxic effect throughout the observation period, irrespective of the LCU used. For the extracts of the Z250 specimens, the cytotoxicity observed was generally higher. |
| **9. Tadin et al. 2013**  **ROB: medium** | - Specimen discs 0.1 g and 0.2 g, height of 2 mm  - Control: Tissue culture plastic | - HGF (3 donors)  - Primary  - Human pulp fibroblasts,  Primary | - 1 brand  - Group:  - Unknown or not provided by the authors (G) | - Viability with acridine orange/ethidium bromide viability staining  - DNA damage with comet assay  - Incubation time 14 days | Significant cytotoxicity was observed for 40 mg/ml of Vertise Flow in both cultures, while Kalore (40 mg/ml) showed cytotoxic effect only on human pulp fibroblasts. A significant level of DNA damage was detected for both materials and concentrations, in both cell cultures. |
| **10. Ajaj et al. 2013**  **ROB: medium** | - Unclear specimen size  - Eluates used🡪 during 2 weeks of leakage  - Control: Tissue culture plastic | - HGF (unknown n donors)  - Unclear primary or cell line | - 14 brands  - Groups:  - BisGMA and TEGDMA (A)  - UDMA+TEGDMA/Bis-GMA/BisEMA (B)  - BisGMA+Bisphenol A dimethacrylate+Bisphenol Amethacrylate, TEGDMA,  BisEMA (E)  - Unknown or not provided by the authors (G) | - Viability with MTT assay  - Incubation time 72 h | It was also found that these resin composite brands possess different leaching abilities with regard to the amount and type of materials and different cytotoxic effects, which were found to be threshold dependent, meaning there is a critical or threshold value of leaching material at or above which the toxic effect will be significant and below which there is no toxic effect. |
| **11. Attik et al. 2014**  **ROB: medium** | - Spherical specimens, diameter 2 mm  - Eluates/extracts used  - Control: Tissue culture plastic | - HGF (unknown n donors)  - Primary | - 1 brand  - Group:  - BisGMA+BisphenolA glyceralate dimethacrylate / Bis-GMA, triethylene glycol dimethacrylate / Bis-GMA, Hydrophobic aromatic dimethacrylate (C) | - Cytotoxicity via microscopy staining  - Time-lapse confocal imaging (live-dead staining) | Image analysis showed a very slight cytotoxicity in the presence of the tested composite after 5 hours of time lapse. A slight decrease of cell viability was shown in contact with the tested composite extracts compared to control cells. |
| **12. Schulz et al. 2015**  **ROB: medium** | - Specimen discs 7 mm diameter, 2 mm height  - Disinfection with 75% ethanol for 60 s  - Eluates of 24h and 7 days  - Control: Tissue culture plastic | - HGK  Immortalized  - HGF (unknown n donors)  - As separate monocultures  And as cocultures | - 3 brands  - Groups:  - BisGMA+BisphenolA glyceralate dimethacrylate / Bis-GMA, triethylene glycol dimethacrylate / Bis-GMA, Hydrophobic aromatic dimethacrylate (C)  - BisGMA+Bisphenol A dimethacrylate+Bisphenol Amethacrylate, TEGDMA,  BisEMA (E)  - Unknown or not provided by the authors (G) | - Apoptosis (Annexin V)  immunofluorescence  - Gene expression qPCR, genes: CASP 3, 8, 9; MKI 67, IL 6, 18, ANX A5, INV, FLG, ITGB3  - Incubation time 24h and 7 days | HGF cultures displayed lower transcription for cell adhesion markers in both cell systems. Filtek Supreme XT showed increased differentiation by elevated filaggrin gene expression in both cell systems for IHGK at day 7, while Filtek Silorane and Ceram X yielded elevation of inflammation biomarkers in both cell types. Annexin-V labeling revealed high apoptosis rates for both flowables and Filtek Supreme XT for IHGK, while low rates were detected for Filtek Silorane and Ceram X. |
| **13. Attik et al. 2017**  **ROB: medium** | - Specimen discs 2 mm diameter, unknown height  - Eluates used  - Control: Tissue culture plastic | - HGF (unknown n donors)  - Primary | - 3 brands and 6 experimental resin composites  - Groups:  - UDMA+TEGDMA/Bis-GMA/BisEMA (B)  - BisGMA+BisphenolA glyceralate dimethacrylate / Bis-GMA, triethylene glycol dimethacrylate / Bis-GMA, Hydrophobic aromatic dimethacrylate (C)  - BisGMA+Bisphenol A dimethacrylate+Bisphenol Amethacrylate, TEGDMA,  BisEMA (E) | - Confocal microscopy  Time-lapse photo every hour for 5 h  - Incubation time 24 h  Outcome:  - Viability  - Cytotoxicity (Calcein AM staining) | Experimental samples were better tolerated than tested commercial samples not containing TEGDMA and significantly better than those containing TEGDMA. Experimental composites with porous fillers exhibited good cytocompatibility, especially when surfaces were silanated. Cytotoxicity was associated with resin amount and especially resin nature. Composites containing porous fillers might behave as if the resin trapped into pores has no effect on toxicity. The cytotoxicity of composites with and without BPA derivatives was mainly attributed to the release of residual TEGDMA rather than the BPA derivatives. |
| **14. Koohpeima et al. 2017**  **ROB: medium** | - Specimen discs 5 mm in diameter, 2 mm height  - Sterilization at 170°C for one hour  - Eluates used  - Control: Tissue culture plastic | - HGF  - Cell line | - 1 brand  - Group:  - BisGMA+BisphenolA glyceralate dimethacrylate / Bis-GMA, triethylene glycol dimethacrylate / Bis-GMA, Hydrophobic aromatic dimethacrylate (C)  - BisGMA+Bisphenol A dimethacrylate+Bisphenol Amethacrylate, TEGDMA,  BisEMA (E) | - Cytotoxicity and viability with MTT assay  - Incubation time 5 h | The materials evaluated on HGF cells, showed significantly more cytotoxicity in silver reinforced glass ionomer but nanohybrid composite shows mild cytotoxic effect. |
| **15. Frese et al. 2018**  **ROB: medium** | - Specimen discs, 5 mm diameter and 1.7 mm height  - Surface either untreated or aluminum blasted with 50um or 110um  - Leached-out by washing for 10 min  - Control: tissue culture plastic | - HGF (unknown n donors)  - Primary cells  - Human Osteoblast cells  - Cell line hFOB1.19 | - 3 brands  - Groups:  - UDMA+TEGDMA/Bis-GMA/BisEMA (B)  - ORMOCER® (F) | - Measuring the monomer release after 1 hour , 1, 7 with high-performance liquid chromatography.  - Cell adhesion measured with MTT assay  - Surface texture of RBC measured with SEM  - Cellular morphology measured after 24h incubation with fluorescent microscopy | As compared with tissue culture plastics, cellular adhesion was significantly lower for human gingival fibroblasts and osteoblasts. Only minor, nonsignificant differences between individual RBCs were identified. The present findings suggest that surface adhesion in the subgingival area is limited for the tested RBCs. |
| **16. Goncalves et al. 2018**  **ROB: medium** | - Specimen discs 5 mm diameter 1 mm height  - Specimens immersed in medium for 24h  - Eluates used  - A2 shade  - Control: tissue culture plastic | - HGF (unknown n donors)  - Primary cells | - 1 brand  - Group:  - BisGMA+Bisphenol A dimethacrylate+Bisphenol Amethacrylate, TEGDMA,  BisEMA (E) | - Measures the degree of conversion using Fourier transform infrared  - Volumetric shrinkage measured with strain gauge method  - Cytotoxicity measured with MTT assay  - Incubation time 24h | All materials, including bulk-fill and conventional composites, were classified as  non-toxic, with cell viability higher than 70%. |
| **17. Hussain et al. 2017**  **ROB: medium** | - Specimens 2mm (± 1mm), weight 76,6 ± 3.6 mg) shape unknown  - Stored in acetone to measure elution after 24 h  - Eluates is used  - Control: Tissue culture plastic | - HGF  - Unclear cell line or primary  - Epithelial cell line A549 (epithelial lung carcinoma cell line) | - 2 brand  - Group:  - UDMA+TEGDMA/Bis-GMA/BisEMA (B)  - BisGMA+Bisphenol A dimethacrylate+Bisphenol Amethacrylate, TEGDMA,  BisEMA (E) | - Outcomes: monomer elution, filler content, hardness, morphology of fillers and biocompatibility and cytotoxicity  - Cytotoxicity measured with LDH  - Incubation time 24 and 48 hours | The RBC outperformed conventional composite regarding mechanical characteristics (hardness) and monomer elution but showed some worrisome results regarding cytotoxicity. |
| **18. Yang et al. 2017**  **ROB: medium** | - Specimen discs 10 mm diameter, 2 mm height  - Eluates used after 24h leakage  - Control: Tissue culture plastic | - HGF  - Nuclear primary or cell line | - 6 brands  - Groups:  - BisGMA and TEGDMA (A)  - UDMA+TEGDMA/Bis-GMA/BisEMA (B)  - BisGMA+Bisphenol A dimethacrylate+Bisphenol Amethacrylate, TEGDMA,  BisEMA (E)  - ORMOCER® (F) | - Viability with XTT-based assay; incubation time 24 h  - DNA damage with immunofluorescence; incubation time 6 h | HGFs exposed to the eluates of all investigated composites showed no significant loss of cell viability, compared to negative control. Significant DNA-DSBs induction could be found in HGFs exposed to the eluates of Esthet.X®HD (0.43 ± 0.05 foci/cell) and Venus®(0.39 ± 0.04 foci/cell), compared to control (0.22 ± 0.03 foci/cell). Significant DNA-DSBs induction can be found in HGFs exposed to the eluates of Esthet.X®HD and Venus®. The interactive effects among released (co)monomers and additives may influence the cytotoxicity and induction of DNA-DSBs, compared to exposure with single composite component. |
| **19. Jerg et al. 2017**  **ROB: medium** | - Specimen discs 6 mm in diameter, 2 mm height  - Disinfection with 75% ethanol for 60 s  - Stored in medium  In a dark box  - Eluates of 24h, 72h used  - Control: Tissue culture plastic | - Human gingival keratinocytes (HGK) immortalized  - Human gingival Fibroblasts (HGF) (unknown n donors) | - 3 brands  - Groups:  - BisGMA+Bisphenol A dimethacrylate+Bisphenol Amethacrylate, TEGDMA,  BisEMA (E)  - Unknown or not provided by the authors (G) | - Cell impendence analysis, continuous measurement for 72h  - Protein expression, indirect immunofluorescence and ELISA, incubation time 24 and 72 h | Generally, cell behavioral response towards the eluates was gradual in HGK and HGF, the latter exhibiting a less pronounced modulation per se. In detail, protein expression and secretion is modulated particularly in terms of signal transduction, differentiation and inflammation. On cell biological level, all tested materials modulated the analyzed features of cell behavior with emphasis on the self-adhering composites. |
| **20. Johnsen et al.**  **2017**  **ROB: medium** | - Specimen for cytotoxicity: 25x2x2 mm  - Samples were rinsed with deionized water  - Eluates were used for other analysis  - Shade A3  - Control: Tissue culture plastic | - A549 cells (human epithelial, lung carcinoma cell line  - HGF (unknown n donors)  - Primary human osteoblasts | - 3 brands  - Groups:  - BisGMA+Bisphenol A dimethacrylate+Bisphenol Amethacrylate, TEGDMA,  BisEMA (E)  - Unknown or not provided by the authors (G) | - Biocompatibility and cytotoxic analyses  - Incubation time 24 h  - Cytotoxicity measured with LDH  - Cell morphology and visualization with microscopy | Statistically significantly higher cytotoxicity combined with altered morphology and loss of confluence was detected in the cells exposed to extracts from the OBLs. |
| **21. Liu et al. 2018**  **ROB: medium** | - Specimen discs diameter 15 mm height 1 mm  - Sterilized with UV radiation for 30 min  - Eluates were used  - Control: Tissue culture plastic | - HGF  - Unclear cell line/primary | - 5 brands  - Groups:  - BisGMA and TEGDMA (A)  - BisGMA+Bisphenol A dimethacrylate+Bisphenol Amethacrylate, TEGDMA,  BisEMA (E) | - Viability measured by fluorescence Cell Count kit-8  - Incubation 72 h  - Intracellular ROS levels  - Mitochondrial functions  Activity of complex IV  - ATP levels  - Mitochondrial membrane potential  - Key regulators of mitochondrial biogenesis  - Mitochondrial morphology | Overall, TEGDMA could elute easier from those resin composites with less filler content and cause oxidative stress in HGFs via mitochondria dysregulation.. |
| **22. Schubert et al. 2018**  **ROB: medium** | - Specimen discs diameter 6 mm height 2 mm  - Shade A3  - Control tissue culture plastic | - L929 mouse dermal fibroblast cell line  - HGF, primary acquired and then immortalized | - 3 brands  - Groups:  - BisGMA+Bisphenol A dimethacrylate+Bisphenol Amethacrylate, TEGDMA,  BisEMA (E)  - ORMOCER® (F) | - Cell viability with CCK-8 assay (cell counting kit 8)  L929 48 h incubation  HGF 72 h incubation | Admira Fusion was significantly less cytotoxic than GrandioSO and Filtek Supreme XTE to both the standard mouse  dermal fibroblasts (L929) and human gingival fibroblasts. |
| **23. Celik et al. 2019**  **ROB: medium** | - Specimen discs, diameter 10 mm and height of 2 mm  - Shade A2  - 3 stages of polishing with water cooling  - Storage of specimens in artificial saliva for 7 and 21 days  - Eluates used  - Control tissue culture plastic | - HGF (unknown n donors)  - Primary cells | - 1 brand  - Group:  - BisGMA+Bisphenol A dimethacrylate+Bisphenol Amethacrylate, TEGDMA,  BisEMA (E) | - Measuring the membrane integrity with Neutral Red uptake test  - Oxidative stress with Total - Antioxidant Capacity (TAC) and Total Oxidant Status (TOS) assay kits  - Incubation time 7 days  Control: no specimen | All tested materials led to significant decreases in the cell viability rates (33–73%) compared to the control group. Glass ionomer and resin composite were found to be more cytotoxic than amalgam alloy and compomer. Increases in TAC levels after seven-day aging in all groups exhibited significant differences with freshly prepared samples. In all material groups, TOS levels of freshly prepared samples differed statistically and significantly from samples aged for 7 and 21 d. |
| **24. Fujioka-Kobayashi et al. 2019**  **ROB: medium** | - Specimen discs 5 mm in diameter, 2 mm height  5 different radiant exposures  - No sterilization with intend  - 2 different light curing unit (LCU) used  - Eluates used  - Control: Tissue culture plastic  - Partly supported by GmcH, Hanau, Germany | - HGF (unknown n donors)  - Primary cells | - 2 brands  - Groups:  - BisGMA/BisEMA+ TEGDMA (A)  - UDMA+TEGDMA/Bis-GMA/BisEMA (B) | - Cell attachment 24h incubation,  - Viability 24h incubation MTS assay,  - PCR cells gown on materials after elution, 24h and cells cultured with eluates for 24h, Elisa cells grown on specimens after elution and cells cultured with eluates, incubation time 3d for both  - PCR genes: IL-1. IL-6, IL-8, TNF-a, Integrin B3, Ki-67, TGF-B, PDGF, ADM, IL11, Caspase 3, Caspase 8, Caspase 9, Anexxin A5, B-actin | Both RBCs light-cured with the T2W at higher REs resulted in higher DC, increased cell adhesion and decreased eluate cytotoxicity. RBC-V induced greater cell adhesion, lower mRNA levels of pro-inflammatory markers, and higher mRNA levels of a proliferation marker than RBC-P. Wettability was the same for both RBCs. Toxicity decreased with increasing number of elution cycles. The initial eluates from RBC-P had a lower toxicity than from RBC-V. |
| **25. Kavuncu et al. 2020**  **ROB: medium** | - Specimen discs diameter 7 mm, height 2 mm  - Incubation time 24h and 1 week  - Control tissue culture plastic | - HGF  - Cell line  - Human periodontal ligament fibroblast  - Cell line | - 3 brands  - Groups:  - BisGMA/BisEMA+ TEGDMA (A)  - UDMA+TEGDMA/Bis-GMA/BisEMA (B)  - ORMOCER® (F) | - Cytotoxicity and viability measured with MTT assay | The cytotoxicity of CRs on hGF and hPDLF cells vary, according to the type of composites, cell types, and exposure time. |
| **26. Alamoush et al. 2020**  **ROB: medium** | - Specimen blocks 5x5x5 mm  - Polished and cleaned in ultrasonic bath  - Sterilization with UV 1h top and 1h bottom  - Timepoints: 1, 3, 5, 10 days  - Control: Tissue culture plastic | - Human gingival fibroblasts (HGF)  - Human gingival keratinocytes (HGK) | - 1 brand  - Group:  - BisGMA+Bisphenol A dimethacrylate+Bisphenol Amethacrylate, TEGDMA,  BisEMA (E) | - Cytotoxicity measured by LDH assay  - Cell viability measured with Alamar Blue  - Cell morphology with light fluorescence microscopy imaging  - Incubation timepoints: 1, 3, 5, 7, 10 days  - Magnification 10x | There was significant difference in cell proliferation of the HGK and HGF cells in contact with different composite materials but no significant differences in their cytotoxicity. There was a significant effect on cell proliferation and cytotoxicity with different exposure times, for each type of resin-composite. HGK proliferation and cytotoxicity appeared to be more influenced by composite materials compared to HGF, demonstrating EN cytotoxic effects in HGK. |
| **27. Haugen et al. 2020**  **ROB: medium** | - Specimen discs diameter 4 mm, height 10 mm  - Eluates used after leakage of 24h with comparable amounts as saliva wash  - Shade A3  - Control: Tissue culture plastic | - A549 cell line epithelial lung carcinoma  - HGF  - Unclear primary/cell line  - Human osteoblasts  Unclear primary/cell line | - 1 brand  - Group:  - UDMA+TEGDMA/Bis-GMA/BisEMA (B) | - Cytotoxicity with LDH  - Cell morphology with microscopy  - Incubation time 24 h | Conditioned media accumulated for 24 h from FBF and TEC were cytotoxic to primary human osteoblasts. |
| **28. Ilie et al. 2021**  **ROB: medium** | - Specimen discs diameter 5 mm, height 2 mm  - Eluates, collected every 24 h for 3 days  - Shade A3  - Control: cells in culture medium in tissue culture plastic | - HGF  - Primary cells | - 2 brands  - Group:  - BisGMA+ TEGDMA (A)  - UDMA +TEGDMA (B) | - Viability with MTS assay | Toxicity decreased with  radiant exposures (RE) and elution cycles and was  lower for Venus Pearl. Within one RE level, addition of violet light resulted in significantly improved properties  (in 131 out of 150 comparisons, p < 0.05). This effect was stronger for Venus Pearl. There was a good correlation  between mechanical and biological parameters. This correlation decreased as the number of eluates increased. |
| **29. Beltrami et al. 2021**  **ROB: medium** | - Specimen discs diameter 7 mm, height 2 mm  - Eluates for 48 h or 72 h  - Control: cells in culture medium in tissue culture plastic | - HGF  - Cell line ATCC CRL-2014 | - 8 brands  - Groups:  - UDMA+TEGDMA/Bis-GMA/BisEMA (B)  - Bis-GMA, triethylene glycol dimethacrylate (C)  - Urethane dimethacrylate,  tricyclodecane imethanol  dimethacrylate, no comonomers (D)  - ORMOCER® (F) | - Viability with MTT  - In categories:  - Non-cytotoxic: >90% viability.  - Mildly cytotoxic: 60–90% viability.  - Moderately cytotoxic: 30–60% viability.  - Severely cytotoxic: <30% viability | Composite resins that contained lower percentages of unbound free monomers—and that released less ions—possessed superior biocompatibility *in vitro*. |
| **30. Roussou et al. 2021**  **ROB: medium** | - Specimen discs diameter 5 mm, height 2 mm  - Eluates for 24 h or 1 month  - In artificial saliva  - Control: cells in culture medium in tissue culture plastic | - HGF  - Primary | - 1 brand  - UDMA+TEGDMA/Bis-GMA/BisEMA (B) | - Viability with Modified Sulforhodamine B (SRB) Assay | The newly introduced bioactive restorative was found to be more biocompatible. |
| **31. De Angelis et al. 2021**  **ROB: medium** | - Specimen discs diameter 5 mm, height 2 mm  - Shade A2  - Eluates for 24 h and 14 d  - Control: cells in culture medium in tissue culture plastic | - HGF  - Primary | - 5 brands  - UDMA +TEGDMA (B)  - BisGMA+BisphenolA glyceralate dimethacrylate / Bis-GMA, triethylene glycol dimethacrylate / Bis-GMA, Hydrophobic aromatic dimethacrylate (C)  - Urethane dimethacrylate,  tricyclodecane imethanol  dimethacrylate (D)  - BisGMA+Bisphenol A dimethacrylate+Bisphenol Amethacrylate, TEGDMA,  BisEMA (E) | - Viability with MTT assay  - Oxidative stress with ROS Flow Cytometry Analysis  - DNA damage with Immunofluorescence for γH2AX expression and visualized with Confocal Microscopy | Simply excluding one specific monomer from the CR formulation might not steadily turn out as a successful approach for improving their biocompatibility. |
| **32. Hoseinifar et al. 2022**  **ROB: medium** | - Specimen discs diameter 4 mm, height 2 mm  - Shade A2  - Eluates for 24 h  - Control: cells in culture medium in tissue culture plastic | - HGF  - Dental pulp stem cells  - Primary | - 2 brands  - Urethane dimethacrylate,  tricyclodecane imethanol  dimethacrylate (D)  - BisGMA+Bisphenol A dimethacrylate+Bisphenol Amethacrylate, TEGDMA,  BisEMA (E)  - ORMOCER® (F) | - Viability with MTT assay and Neutral Red (NR) Assay (Lysosomal Activity)  - Cytotoxicity with Flow Cytometry (Apoptosis and Necrosis)  - Gene expression for apoptosis with Quantitative Real-Time PCR (RT-PCR) | The results of MTT test: Grandio and X‑trafil showed a significant cytotoxicity. In NR-test, Grandio and X‑trafil composites showed a significant cytotoxicity.  RT‑PCR: bax gene expression in the Grandio composite was significant.  All of the tests  showed no significant toxicity of Admira fusion. |
| **33. Aydın et al. 2023**  **ROB: medium** | - Specimen 12x8x2 mm^3^  - Shade A2  - Eluates for 24 h  - Control: cells in culture medium in tissue culture plastic | - HGF  - L929 (Mouse Fibroblast)  - Primary | - 1 brand  - Urethane dimethacrylate,  tricyclodecane imethanol  dimethacrylate (D)  - BisGMA+Bisphenol A dimethacrylate+Bisphenol Amethacrylate, TEGDMA,  BisEMA (E) | - Viability with MTT assay  - Cytotoxicity = viability lower than 70%  - After 24h and 72h  - Qualitative Morphological Analysis after 72h | 3D-printed permanent restoration resins showed similar cell viability on HGF-1 and L929 cells to  resin-based CAD/CAM blocks and composite resin. |
| **34. Ghezzi et al. 2024**  **ROB: Low** | - Specimen discs unclear dimensions  - Shade A2  - Eluates for 1 d and 10 d  - Control: cells in culture medium in tissue culture plastic | - HGF-1  - Cell line from ATCC | - 1 brand  - UDMA+TEGDMA/Bis-GMA/BisEMA (B) | - Cytotoxicity with indirect cytotoxic Assay  - Cell growth with Cell Proliferation Assay, 1, 3, 7, 14 d  - Gene expression with TR-PCR for apoptosis (Caspase 3), Proliferation (CDK2), inflammatory markers (TGF-B (anti-inflammatory and IL-6 inflammatory) | Surfaces wettability as well as cellular behavior seem to be affected by the selection of the finishing system used to lastly shape the restoration. Especially, the presence of OIL act as a negative factor in the regards of human gingival fibroblasts. |
| **35. Flottes and Dursun 2025**  **ROB: medium** | - Specimen discs diameter 3 mm, height 4 mm  - Eluates for 24 h, 72 h  - Control: cells in culture medium in tissue culture plastic | - HGF  - Primary | - 1 brand  - UDMA+TEGDMA/Bis-GMA/BisEMA (B) | - Proteomic analysis with:  - Bioinformatic analysis using databases like UniProt, STRING, and Gene Ontology (GO) to identify protein interactions and affected pathways  - cell viability with light microscopy (no statistics) | The products released from the resin composite tested led to changes in the fibroblast proteome. Under the conditions of this study, resin composite released products can cause early adverse effects on cells, but without complete inhibition of their cellular functions. |
| **36. Mahmoud et al. 2025**  **ROB: medium** | - Specimen discs diameter 5 mm, height 2 mm  - Eluates for 48 h  - Control: cells in culture medium in tissue culture plastic | - HGF  - Primary | - 2 brands  - UDMA+TEGDMA/Bis-GMA/BisEMA (B)  - BisGMA+BisphenolA glyceralate dimethacrylate / Bis-GMA, triethylene glycol dimethacrylate / Bis-GMA, Hydrophobic aromatic dimethacrylate (C)  - BisGMA+Bisphenol A dimethacrylate+Bisphenol Amethacrylate, TEGDMA,  BisEMA (E) | - Viability with MTT assay  - DNA-damage (cell death) with Annexin-V/propidium iodide staining  - Inflammatory response with RT-qPCR for Interleukin-6 (IL-6) and Interleukin-1β (IL-1β) | The HGF viability was significantly higher in the OMN groups than ESPE groups at most of the concentrations. Total cell death was higher in the ESPE groups and at the OMN group at 72 h in comparison to the control and was higher in the ESPE groups compared to OMN groups. Furthermore, the IL-1β and IL-6 levels in the OMN group at 72 h and in the ESPE groups were higher than the control one. |
| **37. Hashemian et al. 2025**  **ROB: medium** | - Specimen discs diameter 6 mm, height 2 mm  - Shade A2  - Eluates for 24 h  - Control: cells in culture medium in tissue culture plastic | - HGF  - Unclear either primary or cell-line | - 1 brand  - BisGMA+Bisphenol A dimethacrylate+Bisphenol Amethacrylate, TEGDMA,  BisEMA (E) | - Viability with MTT assay | Increasing the distance from the light-curing tip adversely affects composite polymerization and biocompatibility, likely due to insufficient curing and subsequent monomer and nanoparticle release. |
| **38. Erdal and Karadag, 2025**  **ROB: medium** | - Specimen discs diameter 7 mm, height 2 mm  - Eluates  - Control: cells in culture medium in tissue culture plastic | - HGF-1  - Cell line | - 7 brands  - UDMA+TEGDMA/Bis-GMA/BisEMA (B)  - BisGMA+BisphenolA glyceralate dimethacrylate / Bis-GMA, triethylene glycol dimethacrylate / Bis-GMA, Hydrophobic aromatic dimethacrylate (C)  - BisGMA+Bisphenol A dimethacrylate+Bisphenol Amethacrylate, TEGDMA,  BisEMA (E) | - Cytotoxicity with MTT assay  - ROS with FRAP assay to measure Total Antioxidant Capacity (TAC) | In the day-1 cytotoxicity assay, Charisma Diamond One was found to be the most cytotoxic of the resin composites tested and control group. Considering the day 7 results, all the resin composites caused a significant decrease in cell viability compared with that of control group. When the total antioxidant capacity of the cells incubated with the resin composites was compared, the cells incubated with the Charisma Diamond One presented the highest values, whereas the Omnichroma group presented significantly lower values than did the other resin composites tested. |

Table S.1: Overview of the included studies and characteristics processed for data extraction

ROB: risk of bias
