# Supplementary material for: Biological effects of dental resin composites on human gingival fibroblasts: a systematic review of in vitro evidence
Source: Clin Oral Investig. 2026 Oct 5;30(10):510. doi: 10.1007/s00784-026-07149-3 (PMC13638730; doi:10.1007/s00784-026-07149-3)
Supplement: Supplementary file 1 [file 784_2026_7149_MOESM1_ESM.zip › Table S.2.docx]

**Biological Effects of Dental Resin Composites on Human Gingival Fibroblasts: A Systematic Review of *In Vitro* Evidence**

**Elmira Boloori ^1^** (<https://orcid.org/0000-0002-9048-7988>)

**Dagmar E. Slot ^1^** (<https://orcid.org/0000-0001-7234-0037>)

**Bruno G. Loos ^1^** (https://orcid.org/[0000-0002-8794-552X](https://orcid.org/0000-0002-8794-552X))

**Teun J. de Vries ^1^** (<https://orcid.org/0000-0001-6464-5172>)

**G.A. van der Weijden ^1^** (<https://orcid.org/0000-0002-5075-8384>)

**^1^** Department of Periodontology, Academic Centre for Dentistry Amsterdam (ACTA), University of Amsterdam and Vrije Universiteit Amsterdam, Amsterdam, The Netherlands

**Corresponding author:**

E. Boloori,

Academic Centre for Dentistry Amsterdam (ACTA),

University of Amsterdam and Vrije Universiteit Amsterdam

Department of Periodontology

Gustav Mahlerlaan 3004, 1081 LA Amsterdam, The Netherlands

Phone: +(31)20 5980 179

E-mail: e.boloori@acta.nl

Journal: Clinical Oral Investigations

| Group | Content | Composite brands | Authors |
| --- | --- | --- | --- |
| A | BisGMA/BisEMA+ TEGDMA | **AP X** (Clearfil AP-X (Kuraray Dental / Kuraray Noritake), Tokyo, Tokyo, Japan) | Yang et al. 2017, Liu et al. 2018 |
|  |  | **Durafill Vs** (Durafill® VS, Kulzer / Heraeus Kulzer, Hanau, Hesse, Germany) | Sigusch et al. 2007, Ajaj et al. 2013 |
|  |  | **Estelite Quick sigma** (Estelite Σ / Estelite Sigma Quick, Tokuyama Dental, Tokyo, Tokyo, Japan) | Kavuncu et al. 2020 |
|  |  | **Herculite** (Herculite, Kerr Corporation, Brea, California, United States) | Franz et al. 2007, Deb et al. 2011 |
|  |  | **Prisma AP H** (Unclear origins) | Ajaj et al. 2013 |
|  |  | **Venus** (Venus, Heraeus Kulzer, Hanau, Hesse, Germany) | Ajaj et al. 2013, Yang et al. 2017, Fujioka-Kobayashi et al.  2019, Ilie 2021 |
| B | UDMA+TEGDMA/Bis-GMA/BisEMA | **Charisma / Topaz** (Charisma, Topaz Kulzer / Heraeus Kulzer, Hanau, Hesse, Germany) | Willershausen et al. 1999, Kavuncu et al. 2020 |
|  |  | **Charisma Diamond One** (Charisma, Topaz Kulzer / Heraeus Kulzer, Hanau, Hesse, Germany) | Erdal and Karadag, 2025 |
|  |  | **Enamel Plus HRi** (Micerium S.p.A., Avegno (GE), Italy) | Roussou et al. 2021, De Angelis et al. 2021, Beltrami et al. 2021 |
|  |  | **Essentia Universal** (GC Corporation, Tokyo, Japan) | Erdal and Karadag, 2025 |
|  |  | **G-aenial anterior/posterior** (G-aenial GC Corporation / GC America for US, Tokyo, Japan) | Attik et al. 2016, Beltrami et al. 2021, Ghezzi et al. 2024 |
|  |  | **Omnichroma** (Tokuyama Dental Corporation Tokyo, Japan) | Beltrami et al. 2021, Mahmoud et al. 2025, Erdal and Karadag, 2025 |
|  |  | **Quixfil** (QuiXfil, Dentsply Sirona / formerly Dentsply, York, Pennsylvania, United States) | Yang et al. 2017 |
|  |  | **ROK** (ROK composite, SDI Limited, Bayswater / Melbourne, Victoria, Australia) | Ajaj et al. 2013 |
|  |  | **Tetric- Flow/Evo Ceram/ Ceram** (Tetric series, Ivoclar Vivadent, Schaan, Liechtenstein) | Willerhausen et al. 1999, Stanislawski et al. 2000, Ajaj et al. 2013, Attik et al. 2016, Frese et al. 2017, Hussain et al. 2017, Haugen et al. 2020, Flottes, Dursun 2025 |
|  |  | **Venus pearl/diamond** (Venus Pearl, Kulzer / Heraeus Kulzer, Hanau, Hesse, Germany) | Fujioka-Kobayashi et al. 2019, Ilie et al. 2021, De Angelis et al. 2021 |
|  |  | **Vittra APS Unique** (FGM Dental Group, Brazil) | Erdal and Karadag, 2025 |
|  |  | **Zenchroma** (President Dental, Germany) | Erdal and Karadag, 2025 |
| C | BisGMA+BisphenolA glyceralate dimethacrylate / Bis-GMA, triethylene glycol dimethacrylate / Bis-GMA, Hydrophobic aromatic dimethacrylate | **Clearfil Majesty ES-2 Classic** (Kuraray Dental / Kuraray Noritake,Tokyo, Japan) | De Angelis et al. 2021, Erdal and Karadag, 2025 |
|  |  | **ELS** (ELS, Saremco Dental AG, Rebstein, Switzerland) | Attic et al. 2014, Attik et al. 2016 |
|  |  | **Omnichroma Blocker** (Tokuyama Dental Corporation Tokyo, Japan) | Beltrami et al. 2021, Mahmoud et al. 2025, Erdal and Karadag, 2025 |
| D | Urethane dimethacrylate,  tricyclodecane imethanol  dimethacrylate | **Enamel Plus Hri Bio Function Enamel** (Micerium S.p.A., Avegno, Italy) | Beltrami et al. 2021, De Angelis et al. 2021 |
| E | BisGMA+Bisphenol A dimethacrylate+Bisphenol Amethacrylate, TEGDMA, | **Clearfil Majesty posterior** (Kuraray Dental / Kuraray Noritake, Tokyo, Japan) | Aydın et al. 2023 |
|  | BisEMA | **Estet X** (Esthet-X, Dentsply Sirona / Dentsply Caulk, York, Pennsylvania, United States) | Ajaj et al. 2013, Yang et al. 2017 |
|  |  | **Filtek P60** (Filtek P60, 3M Oral Care / 3M ESPE, St. Paul / Maplewood, Minnesota, United States) | Liu et al. 2018, Deb et al. 2011 |
|  |  | **Filtek Supreme XTE/Plus** (Filtek Supreme XTE / Plus, 3M Oral Care, St. Paul / Maplewood, Minnesota, United States) | Ajaj et al. 2013, Schulz et al. 2015, Frese et al. 2017, Jerg et al. 2017 |
|  |  | **Filtek Z250** (Filtek Z250, 3M Oral Care / 3M ESPE, St. Paul / Maplewood, Minnesota, United States) | Sigusch et al. 2007, Sigusch et al. 2012, Attik et al. 2016, Koohpeima et al. 2017  Hussain et al. 2017, Johnsen et al. 2017, Celik et al. 2019 |
|  |  | **Filtek Z350** (Filtek Z350, 3M Oral Care / 3M ESPE, St. Paul / Maplewood, Minnesota, United States) | Liu et al. 2018, Goncalves et al. 2018, Mahmoud et al. 2025, Hashemian et al. 2025 |
|  |  | **Grandio** / SO (Grandio, VOCO GmbH, Cuxhaven, Lower Saxony, Germany) | Ajaj et al. 2013, Sigusch et al. 2007, Sigusch et al. 2012, Schubert et al. 2018, Alamoush et al. 2020, De Angelis et al. 2021, Hoseinifar et al. 2022 |
|  |  | **Heliomolar** (Heliomolar, Ivoclar Vivadent, Schaan, Liechtenstein) | Ajaj et al. 2013, Deb et al. 2011 |
|  |  | **Spectrum TPH** (Spectrum TPH, originally by Dentsply/Caulk, York, Pennsylvania, United States) | Huang et al. 2002, Deb et al. 2011, Ajaj et al. 2013 |
|  |  | **TopDent** (Unclear origins) | Johnsen et al. 2017 |
|  |  | **Vita Zeta** (VITA, VITA Zahnfabrik, Bad Säckingen, Baden-Württemberg, Germany) | Liu et al. 2018 |
|  |  | **X-tra fil** (VOCO GmbH, Cuxhaven, Lower Saxony, Germany) | Erdal and Karadag, 2025 |
| F | ORMOCER® | **Admira / fusion** (Admira Fusion VOCO, Cuxhaven, Lower Saxony, Germany) | Frese et al. 2018, Yang et al. 2017, Schubert et al. 2018, Kavuncu et al. 2020, Beltrami et al. 2021, Hoseinifar et al. 2022 |
| G | Unknown/not provided by author | **3D-direct** (Unclear origins) | Ajaj et al. 2013 |
|  |  | **4 Seasons** (Unclear origins) | Ajaj et al. 2013 |
|  |  | **4U** (Unclear origins) | Johnsen et al. 2017 |
|  |  | **Ceram X** (Ceram.X, Dentsply Sirona / formerly Dentsply DeTrey, York, Pennsylvania, United States) | Schulz et al. 2015, Jerg et al. 2017 |
|  |  | **Filtek Silorane** (Filtek Silorane, 3M ESPE, St. Paul / Maplewood, Minnesota, United States | Schulz et al. 2015, Jerg et al. 2017 |
|  |  | **ICE** (ICE composite, SDI Limited, Bayswater / Melbourne, Victoria, Australia) | Ajaj et al. 2013 |
|  |  | **Kalore** (Kalore, GC America, Tokyo, Japan) | Tadin et al. 2013 |
|  |  | **Multifil** (Unclear origins) | Caughman et al. 1991 |
|  |  | **P50** (Unclear origins) | Caughman et al. 1991 |
|  |  | **Prisma-micro-fine** (Unclear origins) | Caughman et al. 1991 |
|  |  | **Quixx** (Unclear origins) | Ajaj et al. 2013 |
|  |  | **Solitair / 2** (Unclear origins) | Willerhausen et al. 1999, Sigusch et al. 2007, Sigusch et al. 2012 |
|  |  | **Sure fill** (Unclear origins) | Ajaj et al. 2013 |
|  |  | **Z100 MP** (Z100, 3M ESPE dental composite line, St. Paul / Maplewood, Minnesota, United States) | Stanislawski et al. 2000 |

Table S.2: Resin composite brands sorted into 7 groups (A-G) based on their content
