# Supplementary material for: Biological effects of dental resin composites on human gingival fibroblasts: a systematic review of in vitro evidence
Source: Clin Oral Investig. 2026 Oct 5;30(10):510. doi: 10.1007/s00784-026-07149-3 (PMC13638730; doi:10.1007/s00784-026-07149-3)
Supplement: Supplementary file 1 [file 784_2026_7149_MOESM1_ESM.zip › Table S.4.docx]

**Biological Effects of Dental Resin Composites on Human Gingival Fibroblasts: A Systematic Review of *In Vitro* Evidence**

**Elmira Boloori ^1^** (<https://orcid.org/0000-0002-9048-7988>)

**Dagmar E. Slot ^1^** (<https://orcid.org/0000-0001-7234-0037>)

**Bruno G. Loos ^1^** (https://orcid.org/[0000-0002-8794-552X](https://orcid.org/0000-0002-8794-552X))

**Teun J. de Vries ^1^** (<https://orcid.org/0000-0001-6464-5172>)

**G.A. van der Weijden ^1^** (<https://orcid.org/0000-0002-5075-8384>)

**^1^** Department of Periodontology, Academic Centre for Dentistry Amsterdam (ACTA), University of Amsterdam and Vrije Universiteit Amsterdam, Amsterdam, The Netherlands

**Corresponding author:**

E. Boloori,

Academic Centre for Dentistry Amsterdam (ACTA),

University of Amsterdam and Vrije Universiteit Amsterdam

Department of Periodontology

Gustav Mahlerlaan 3004, 1081 LA Amsterdam, The Netherlands

Phone: +(31)20 5980 179

E-mail: e.boloori@acta.nl

Journal: Clinical Oral Investigations

| **Determinants of the Quality** | **Cytotoxicity (necrosis/apoptosis)** | | | **Viability** | | | | **Proliferation**  **/ Adhesion** | | | **Inflammatory markers** | | | **DNA damage/ modification** | | | **ROS** | | | **Cell**  **morphology** | | | **Cell**  **Attachment** | | |
| --- | --- | --- | --- | --- | --- | --- | --- | --- | --- | --- | --- | --- | --- | --- | --- | --- | --- | --- | --- | --- | --- | --- | --- | --- | --- |
| Study design | …*In vitro* experimental research… | | | | | | | | | | | | | | | | | | | | | | | | |
| # studies (ref.) | N=13 [2-15] | | | N=24 [3, 7, 10, 11, 13-33] | | | | N=2 [15, 34] | | | N=7 [6, 15, 34-38] | | | N=4 [19, 24, 31, 38] | | | N=4 [23, 25, 31, 33] | | | N=8 [8, 9, 12, 21, 25, 35, 36, 39] | | | N=6 [3, 6, 9, 35, 36, 39] | | |
| # composite (group) | N=6 (A, B, C, E, F, G) | | | N=7 (A, B, C, D, E, F, G) | | | | N=3 (B, E, G) | | | N=5 (A, B, C, E, G) | | | N=7 (A, B, C, D, E, F, G) | | | N=4 (B, C, D, E) | | | N=4 (B, C, D, F) | | | N=4 (B, E, F, G) | | |
| # variables e.g. composites (C), biological testing (A) | Ref. | #C | #A | Ref. | #C | | #A | Ref. | #C | #A | Ref. | #C | #A | Ref. | #A | #A | Ref. | #C | #A | Ref. | #C | #A | Ref. | #C | #A |
|  | [2] | 3 | 1 | [16] | 2 | | 2 | [34] | 2 | 2 | [34] | 2 | 2 | [5] | 1 | 2 | [23] | 1 | 1 | [12] | 1 | 2 | [3] | 1 | 1 |
|  | [3] | 1 | 1 | [3] | 1 | | 1 | [15] | 1 | 3 | [6] | 3 | 2 | [24] | 6 | 2 | [9] | 5 | 6 | [21] | 1 | 1 | [6] | 3 | 2 |
|  | [4] | 1 | 1 | [17] | 4 | | 1 |  |  |  | [35] | 3 | 2 | [31] | 5 | 3 | [31] | 5 | 3 | [39] | 3 | 3 | [39] | 3 | 3 |
|  | [5] | 1 | 2 | [18] | 3 | | 2 |  |  |  | [36] | 2 | 3 | [38] | 2 | 3 | [33] | 7 | 2 | [8] | 2 | 1 | [35] | 3 | 2 |
|  | [6] | 3 | 2 | [19] | 3 | | 2 |  |  |  | [15] | 1 | 3 |  |  |  |  |  |  | [35] | 3 | 2 | [9] | 2 | 3 |
|  | [7] | 3 | 2 | [20] | 14 | | 1 |  |  |  | [37] | 1 | 1 |  |  |  |  |  |  | [9] | 3 | 2 | [36] | 2 | 3 |
|  | [8] | 2 | 1 | [21] | 1 | | 1 |  |  |  | [38] | 2 | 3 |  |  |  |  |  |  | [36] | 2 | 3 |  |  |  |
|  | [9] | 3 | 2 | [7] | 3 | | 2 |  |  |  |  |  |  |  |  |  |  |  |  | [25] | 1 | 3 |  |  |  |
|  | [10] | 3 | 1 | [22] | 1 | | 1 |  |  |  |  |  |  |  |  |  |  |  |  |  |  |  |  |  |  |
|  | [11] | 1 | 3 | [23] | 1 | | 1 |  |  |  |  |  |  |  |  |  |  |  |  |  |  |  |  |  |  |
|  | [12] | 1 | 2 | [24] | 6 | | 2 |  |  |  |  |  |  |  |  |  |  |  |  |  |  |  |  |  |  |
|  | [13] | 2 | 3 | [25] | 5 | | 6 |  |  |  |  |  |  |  |  |  |  |  |  |  |  |  |  |  |  |
|  | [15] | 1 | 3 | [26] | 3 | | 1 |  |  |  |  |  |  |  |  |  |  |  |  |  |  |  |  |  |  |
|  |  |  |  | [27] | 1 | | 3 |  |  |  |  |  |  |  |  |  |  |  |  |  |  |  |  |  |  |
|  |  |  |  | [10] | 3 | | 1 |  |  |  |  |  |  |  |  |  |  |  |  |  |  |  |  |  |  |
|  |  |  |  | [11] | 1 | | 3 |  |  |  |  |  |  |  |  |  |  |  |  |  |  |  |  |  |  |
|  |  | | | [28] | 2 | 1 | |  | | |  | | |  | | |  | | |  | | |  | | |
|  |  | | | [29] | 8 | 1 | |  | | |  | | |  | | |  | | |  | | |  | | |
|  |  | | | [14] | 1 | 2 | |  | | |  | | |  | | |  | | |  | | |  | | |
|  |  | | | [30] | 1 | 1 | |  | | |  | | |  | | |  | | |  | | |  | | |
|  |  | | | [31] | 5 | 3 | |  | | |  | | |  | | |  | | |  | | |  | | |
|  |  | | | [38] | 2 | 3 | |  | | |  | | |  | | |  | | |  | | |  | | |
|  |  | | | [32] | 1 | 1 | |  | | |  | | |  | | |  | | |  | | |  | | |
|  |  | | | [33] | 7 | 2 | |  | | |  | | |  | | |  | | |  | | |  | | |
|  |  | | | [13] | 2 | 3 | |  | | |  | | |  | | |  | | |  | | |  | | |
| Risk of bias (Supp. table 3) | Medium | | | Medium | | | | Medium | | | Medium | | | Medium | | | Medium | | | Medium | | | Medium | | |
| Consistency | Rather inconsistent | | | Inconsistent | | | | Not applicable (n=2) | | | Inconsistent | | | Inconsistent | | | Consistent | | | Rather inconsistent | | | Inconsistent | | |
| Directness | Not  generalizable | | | Not  generalizable | | | | Not  generalizable | | | Not  generalizable | | | Not generalizable | | | Not generalizable | | | Not  generalizable | | | Not  generalizable | | |
| Precision (based on statistics e.g. SD, Sig., Fig. descript.) | Rather  precise | | | Rather  precise | | | | Imprecise | | | Rather  precise | | | Rather  imprecise | | | Precise | | | Rather  precise | | | Precise | | |
| Reporting bias | Possible | | | Possible | | | | Possible | | | Possible | | | Possible | | | Possible | | | Possible | | | Possible | | |
| Strength of the evidence for the observation based on the quality and body of evidence | Moderate | | | Moderate | | | | Very low | | | Low | | | Very low | | | Moderate | | | Moderate | | | Low | | |
| Overall rating of the evidence | With moderate certainty, there appear to be some effects on cytotoxicity; however, the use of various types of composites is not discouraged based on this outcome measure. Considering the fair consistency of results and the fact that 73% of the experiments show no significant effect of resin composites on gingival fibroblast cells, the evidence suggests that these materials may generally pose a low risk for impacting gingival fibroblast cell health adversely. | | | With moderate certainty, there appear to be some effect on viability; however, the use of various types of composites is not discouraged based on this outcome measure. Considering the inconsistency of results and the fact that 55% of the experiments show no significant effect of resin composites on gingival fibroblast cells, it suggests that these materials may pose some risk for viability of gingival fibroblast. | | | | There is insufficient data to rate the evidence | | | With low certainty, resin composites appear to have a negative effect on inflammation, Considering the poor consistency of results, it is possible that these materials may generally pose a risk for impacting gingival fibroblast cell health adversely and it is advisable to be mindful of this effect. | | | With very low certainty, there is some effect of resin composites on gene toxicity/ modification; however, the use of these materials is not discouraged, as there is a lack of evidence that resin composite has a negative effect on DNA of gingival fibroblast. | | | With moderate certainty, in line with inflammatory markers, resin composites appear to lead to increased reactive oxygen species (ROS) production, and this aspect should be considered. | | | With moderate certainty, resin composites do not appear to have any effect on cell morphology. | | | With low certainty, the attachment of gingival fibroblasts to resin composites appears to be impaired, which may lead to deepening of periodontal pockets in susceptible patients. Therefore, this unfavorable aspect needs to be heeded. | | |
| Overall recommendation | There is considerable contradiction among the reviewed studies, therefore, the strength of the recommendations ranges from very low to moderate. The use of resin composites is not discouraged, as no significant differences have been observed among various types. However, given that resin composites may induce some degree of inflammation, special attention should be given to patients susceptible to gingivitis and periodontitis. Furthermore, there is a critical need for standardization in assessing biocompatibility, ensuring that outcome measures are comprehensive and that consistent protocols and assays are employed. | | | | | | | | | | | | | | | | | | | | | | | | |

Table S.4: Grading of body of evidence

Summary of findings on body of the estimated evidence profile [1] and appraisal of certainty and the strength of the evidence of the observation regarding the effect of various resin composites on gingival fibroblast cells on the parameters of interest.

#C: number of different resin composite brands

#A: number of different assays used

The colors indicate the risk of bias red: substantial risk of bias, orange: moderate risk of bias, green: low risk of bias.

Literature

1.	G.H. Guyatt, A.D. Oxman, R. Kunz, R. Jaeschke, M. Helfand, A. Liberati, G.E. Vist, H.J. Schunemann, G.W. Group (2008) Incorporating considerations of resources use into grading recommendations. BMJ 7654:1170-3. <https://www.ncbi.nlm.nih.gov/pubmed/18497416>

2.	W.F. Caughman, G.B. Caughman, R.A. Shiflett, F. Rueggeberg, G.S. Schuster (1991) Correlation of cytotoxicity, filler loading and curing time of dental composites. Biomaterials 8:737-40. <https://www.ncbi.nlm.nih.gov/pubmed/1799649>

3.	F.M. Huang, K.W. Tai, M.Y. Chou, Y.C. Chang (2002) Resinous perforation-repair materials inhibit the growth, attachment, and proliferation of human gingival fibroblasts. J Endod 4:291-4. <https://www.ncbi.nlm.nih.gov/pubmed/12043866>

4.	A. Franz, F. Konig, A. Skolka, W. Sperr, P. Bauer, T. Lucas, D.C. Watts, A. Schedle (2007) Cytotoxicity of resin composites as a function of interface area. Dent Mater 11:1438-46. <https://www.ncbi.nlm.nih.gov/pubmed/17688932>

5.	A. Tadin, D. Marovic, N. Galic, I. Kovacic, D. Zeljezic (2014) Composite-induced toxicity in human gingival and pulp fibroblast cells. Acta Odontol Scand 4:304-11. <https://www.ncbi.nlm.nih.gov/pubmed/23964631>

6.	S.D. Schulz, C. Ruppell, P. Tomakidi, T. Steinberg, F.X. Reichl, E. Hellwig, O. Polydorou (2015) Gene expression analysis of conventional and interactive human gingival cell systems exposed to dental composites. Dent Mater 11:1321-34. <https://www.ncbi.nlm.nih.gov/pubmed/26345997>

7.	N. Attik, F. Hallay, L. Bois, A. Brioude, B. Grosgogeat, P. Colon (2017) Mesoporous silica fillers and resin composition effect on dental composites cytocompatibility. Dent Mater 2:166-174. <https://www.ncbi.nlm.nih.gov/pubmed/27931721>

8.	B. Hussain, M.K.L. Thieu, G.F. Johnsen, J.E. Reseland, H.J. Haugen (2017) Can CAD/CAM resin blocks be considered as substitute for conventional resins? Dent Mater 12:1362-1370. <https://www.ncbi.nlm.nih.gov/pubmed/28951036>

9.	G.F. Johnsen, M.K. Thieu, B. Hussain, E. Pamula, J.E. Reseland, S.P. Lyngstadaas, H. Haugen (2017) Own brand label restorative materials-A false bargain? J Dent 84-98. <https://www.ncbi.nlm.nih.gov/pubmed/27836814>

10.	G. Kavuncu, A.M. Yilmaz, B. Karademir Yilmaz, P. Yilmaz Atali, E.C. Altunok, L. Kuru, O.B. Agrali (2020) Cytotoxicity of Different Nano Composite Resins on Human Gingival and Periodontal Ligament Fibroblast Cell Lines: An In Vitro Study. Biomedicines 3. <https://www.ncbi.nlm.nih.gov/pubmed/32121617>

11.	R.A. Alamoush, E. Kushnerev, J.M. Yates, J.D. Satterthwaite, N. Silikas (2020) Response of two gingival cell lines to CAD/CAM composite blocks. Dent Mater 9:1214-1225. <https://www.ncbi.nlm.nih.gov/pubmed/32561116>

12.	H.J. Haugen, D. Marovic, M. Par, M.K.L. Thieu, J.E. Reseland, G.F. Johnsen (2020) Bulk Fill Composites Have Similar Performance to Conventional Dental Composites. Int J Mol Sci 14. <https://www.ncbi.nlm.nih.gov/pubmed/32698509>

13.	R. Hoseinifar, A. Eskandarizadeh, L. Lashkarizadeh, A. Derakhshani, F. Arjmand (2022) Cytotoxicity effects of nanohybrid, bulk-fill, and ormocer composites on dental pulp stem cells and human gingival fibroblast cells. Dent Res J (Isfahan) 101. <https://www.ncbi.nlm.nih.gov/pubmed/36605150>

14.	N. Aydin, S. Karaoglanoglu, A. Kilic-Suloglu, S. Ozturk (2023) Comparison of the Cytotoxic Effect of 3D-Printed Resins, Resin-based CAD/CAM blocks and Composite Resin. J Clin Exp Dent 12:e984-e990. <https://www.ncbi.nlm.nih.gov/pubmed/38186911>

15.	B. Ghezzi, M. Meglioli, A. Salvaterra Toffoli, G. Mergoni, F. Rossi, M. Manfredi, S. Lumetti, E. Manfredi (2024) Polishing methods for composites restoration: the influence on human gingival fibroblasts behaviour. BMC Oral Health 1:651. <https://www.ncbi.nlm.nih.gov/pubmed/38831398>

16.	L. Stanislawski, E. Soheili-Majd, A. Perianin, M. Goldberg (2000) Dental restorative biomaterials induce glutathione depletion in cultured human gingival fibroblast: protective effect of N-acetyl cysteine. J Biomed Mater Res 3:469-74. <https://www.ncbi.nlm.nih.gov/pubmed/10880090>

17.	B.W. Sigusch, A. Volpel, I. Braun, A. Uhl, K.D. Jandt (2007) Influence of different light curing units on the cytotoxicity of various dental composites. Dent Mater 11:1342-8. <https://www.ncbi.nlm.nih.gov/pubmed/17229462>

18.	S. Deb, L. Di Silvio, H.E. Mackler, B.J. Millar (2011) Pre-warming of dental composites. Dent Mater 4:e51-9. <https://www.ncbi.nlm.nih.gov/pubmed/21145580>

19.	B.W. Sigusch, T. Pflaum, A. Volpel, K. Gretsch, S. Hoy, D.C. Watts, K.D. Jandt (2012) Resin-composite cytotoxicity varies with shade and irradiance. Dent Mater 3:312-9. <https://www.ncbi.nlm.nih.gov/pubmed/22240279>

20.	R. Ajaj, R. Baier, J. Fabiano, P. Bush (2013) Infrared spectroscopic analysis of restorative composite materials' surfaces and their saline extracts. Prog Biomater 1:9. <https://www.ncbi.nlm.nih.gov/pubmed/29470672>

21.	G.N. Attik, K. Gritsch, P. Colon, B. Grosgogeat (2014) Confocal time lapse imaging as an efficient method for the cytocompatibility evaluation of dental composites. J Vis Exp 93:e51949. <https://www.ncbi.nlm.nih.gov/pubmed/25406737>

22.	F. Koohpeima, M.J. Mokhtari, M. Doozandeh, Z. Jowkar, F. Yazdanshenas (2017) Comparison of Cytotoxicity of New Nanohybrid Composite, Giomer, Glass Ionomer and Silver Reinforced Glass Ionomer using Human Gingival Fibroblast Cell Line. J Clin Pediatr Dent 5:368-373. <https://www.ncbi.nlm.nih.gov/pubmed/28872994>

23.	F. Gonçalves, L.M.P. Campos, E.C. Rodrigues-Júnior, F.V. Costa, P.A. Marques, C.E. Francci, R.R. Braga, L.C.C. Boaro (2018) A comparative study of bulk-fill composites: degree of conversion, post-gel shrinkage and cytotoxicity. Braz Oral Res e17.

24.	Y. Yang, F.X. Reichl, J. Shi, X. He, R. Hickel, C. Hogg (2018) Cytotoxicity and DNA double-strand breaks in human gingival fibroblasts exposed to eluates of dental composites. Dent Mater 2:201-208. <https://www.ncbi.nlm.nih.gov/pubmed/29042079>

25.	B. Liu, X. Gan, Y. Zhao, J. Chen, H. Yu, J. Gao, H. Yu (2019) TEGDMA releasing in resin composites with different filler contents and its correlation with mitochondrial mediated cytotoxicity in human gingival fibroblasts. J Biomed Mater Res A 6:1132-1142. <https://www.ncbi.nlm.nih.gov/pubmed/30597721>

26.	A. Schubert, C. Ziegler, A. Bernhard, R. Burgers, N. Miosge (2019) Cytotoxic effects to mouse and human gingival fibroblasts of a nanohybrid ormocer versus dimethacrylate-based composites. Clin Oral Investig 1:133-139. <https://www.ncbi.nlm.nih.gov/pubmed/29603022>

27.	N. Celik, D. Binnetoglu, N. Ozakar Ilday, A. Hacimuftuoglu, N. Seven (2021) The cytotoxic and oxidative effects of restorative materials in cultured human gingival fibroblasts. Drug Chem Toxicol 5:502-507. <https://www.ncbi.nlm.nih.gov/pubmed/31146597>

28.	N. Ilie, G. Schmalz, M. Fujioka-Kobayashi, A. Lussi, R.B. Price (2021) Correlation of the mechanical and biological response in light-cured RBCs to receiving a range of radiant exposures: Effect of violet light. J Dent 103568. <https://www.ncbi.nlm.nih.gov/pubmed/33385531>

29.	R. Beltrami, M. Colombo, K. Rizzo, A. Di Cristofaro, C. Poggio, G. Pietrocola (2021) Cytotoxicity of Different Composite Resins on Human Gingival Fibroblast Cell Lines. Biomimetics (Basel) 2. <https://www.ncbi.nlm.nih.gov/pubmed/33924063>

30.	K. Roussou, A.K. Nikolaidis, F. Ziouti, A. Arhakis, K. Arapostathis, E.A. Koulaouzidou (2021) Cytotoxic Evaluation and Determination of Organic and Inorganic Eluates from Restorative Materials. Molecules 16. <https://www.ncbi.nlm.nih.gov/pubmed/34443499>

31.	F. De Angelis, D. Mandatori, V. Schiavone, F.P. Melito, S. Valentinuzzi, M. Vadini, P. Di Tomo, L. Vanini, L. Pelusi, C. Pipino, P. Del Boccio, C. D'Arcangelo, A. Pandolfi (2021) Cytotoxic and Genotoxic Effects of Composite Resins on Cultured Human Gingival Fibroblasts. Materials (Basel) 18. <https://www.ncbi.nlm.nih.gov/pubmed/34576450>

32.	A. Hashemian, M. Hodjat, M. Behroozibakhsh (2025) Correlation between gingival fibroblast cell viability and degree of conversion of resin composites at different light-curing tip distances. J Oral Biol Craniofac Res 6:1793-1798. <https://www.ncbi.nlm.nih.gov/pubmed/41210521>

33.	B. Erdal, G. Karadag (2025) Evaluation of the total antioxidant capacity and cytotoxicity of single shade universal resin composites. BMC Oral Health 1:1991. <https://www.ncbi.nlm.nih.gov/pubmed/41310562>

34.	B. Willershausen, D. Schafer, A. Pistorius, R. Schulze, W. Mann (1999) Influence of resin-based restoration materials on cytotoxicity in gingival fibroblasts. Eur J Med Res 4:149-55. <https://www.ncbi.nlm.nih.gov/pubmed/10205290>

35.	A. Jerg, S.D. Schulz, P. Tomakidi, E. Hellwig, O. Polydorou (2018) Modulation of gingival cell response towards dental composites. Dent Mater 3:412-426. <https://www.ncbi.nlm.nih.gov/pubmed/29306486>

36.	M. Fujioka-Kobayashi, R.J. Miron, A. Lussi, R. Gruber, N. Ilie, R.B. Price, G. Schmalz (2019) Effect of the degree of conversion of resin-based composites on cytotoxicity, cell attachment, and gene expression. Dent Mater 8:1173-1193. <https://www.ncbi.nlm.nih.gov/pubmed/31174864>

37.	Y. Flottes, E. Dursun (2025) Proteomic Analysis of Fibroblasts Exposed to Resin Composite Release. Proteomics Clin Appl 1:e202400049. <https://www.ncbi.nlm.nih.gov/pubmed/39422137>

38.	M.G. Mahmoud, A. Shamaa, N.M. Bakr, M. El Shahawy (2025) Biological effects of two nano-composite resins on human gingival fibroblast (an in vitro study). J Oral Biol Craniofac Res 5:1029-1035. <https://www.ncbi.nlm.nih.gov/pubmed/40703617>

39.	C. Frese, D. Wolff, T. Kruger, H.J. Staehle, C.J. Lux, R. Erber (2018) Biological evaluation of subgingivally placed direct resin composite materials. J Oral Sci 1:89-96. <https://www.ncbi.nlm.nih.gov/pubmed/29375100>
