# Supplementary material for: Biological effects of dental resin composites on human gingival fibroblasts: a systematic review of in vitro evidence
Source: Clin Oral Investig. 2026 Oct 5;30(10):510. doi: 10.1007/s00784-026-07149-3 (PMC13638730; doi:10.1007/s00784-026-07149-3)
Supplement: Supplementary file 1 [file 784_2026_7149_MOESM1_ESM.zip › Table S.5.docx]

**Biological Effects of Dental Resin Composites on Human Gingival Fibroblasts: A Systematic Review of *In Vitro* Evidence**

**Elmira Boloori ^1^** (<https://orcid.org/0000-0002-9048-7988>)

**Dagmar E. Slot ^1^** (<https://orcid.org/0000-0001-7234-0037>)

**Bruno G. Loos ^1^** (https://orcid.org/[0000-0002-8794-552X](https://orcid.org/0000-0002-8794-552X))

**Teun J. de Vries ^1^** (<https://orcid.org/0000-0001-6464-5172>)

**G.A. van der Weijden ^1^** (<https://orcid.org/0000-0002-5075-8384>)

**^1^** Department of Periodontology, Academic Centre for Dentistry Amsterdam (ACTA), University of Amsterdam and Vrije Universiteit Amsterdam, Amsterdam, The Netherlands

**Corresponding author:**

E. Boloori,

Academic Centre for Dentistry Amsterdam (ACTA),

University of Amsterdam and Vrije Universiteit Amsterdam

Department of Periodontology

Gustav Mahlerlaan 3004, 1081 LA Amsterdam, The Netherlands

Phone: +(31)20 5980 179

E-mail: e.boloori@acta.nl

Journal: Clinical Oral Investigations

| **Biological endpoint** | **Assay (standardized name)** | **Used for outcome measures** | **Number of studies (n)** |
| --- | --- | --- | --- |
| **Cell viability / cytotoxicity / morphology** | MTT assay | Mitochondrial metabolic activity; viability, cytotoxicity, proliferation, or cell attachment | 19 |
|  | LDH release assay | Membrane integrity and cytotoxicity | 5 |
|  | Alamar Blue assay | Cellular metabolic activity and proliferation | 3 |
|  | XTT / MTS / CCK-8 assays | Tetrazolium-based metabolic activity assays | 5 |
|  | Trypan blue exclusion test | Cell viability and cell count | 1 |
|  | Neutral Red uptake assay | Lysosomal integrity and viability | 1 |
|  | Own-developed cytotoxicity assay | RNA and protein synthesis inhibition | 1 |
|  | Microscopy-based cytotoxicity assessment | Morphology-based or staining-based cytotoxicity evaluation | 4 |
|  | modified sulforhodamine B (SRB) assay | Trypan blue: staining the total cellular protein | 1 |
|  | Live–dead staining (Calcein AM, AO/EB, confocal) | Membrane integrity and esterase activity | 4 |
|  | Indirect Cytotoxicity Assay | Quantifying ATP | 1 |
|  | Cell proliferation Assay CellTiter-GLO | Chemiluminescence | 1 |
| **Apoptosis** | Annexin V assay | Early apoptotic cell detection | 1 |
|  | Flow cytometry (apoptosis/cytotoxicity) | Quantitative assessment of cell death pathways | 3 |
|  | Gene expression RT-PCR | BAX-gene, Caspase 3 expression for apoptosis | 2 |
| **Oxidative stress** | Reactive oxygen species (ROS) assays | Intracellular oxidative stress levels | 3 |
|  | Total Antioxidant Capacity / Total Oxidant Status (TAC/TOS) | Redox balance assessment | 2 |
| **Mitochondrial function** | Mitochondrial function assays | ATP levels, membrane potential, complex IV activity, morphology | 1 |
| **DNA damage** | Comet assay | DNA strand breaks | 1 |
|  | DNA damage immunofluorescence | Detection of DNA double-strand breaks | 2 |
|  | Annexin-V/propidium iodide staining | Cell death | 1 |
| **Inflammation / protein expression** | ELISA-based assays | Cytokine or protein secretion | 2 |
|  | Prostaglandin E (PGE) immunoassay | Inflammatory mediator release | 1 |
|  | Protein content quantification | Total cellular protein | 1 |
|  | Bioinformatic analysis | using databases like UniProt, STRING, and Gene Ontology (GO) to identify protein interactions and affected pathways | 1 |
| **Gene expression** | Quantitative PCR (qPCR)/ real time PCR (RT-PCR) | Expression of apoptosis, inflammation, adhesion, and proliferation markers | 5 |
| **Cell behavior / adhesion** | Cell impedance analysis | Real-time cell adhesion and proliferation | 1 |
|  | MTT-based cell attachment assay | Quantitative cell adhesion measurement | 2 |
| **Cellular redox status** | Glutathione (GSH) depletion assay (mBCI fluorescence) | Intracellular antioxidant depletion | 1 |

Table S.5: Biological assays used in the included studies and their frequency of occurrence
