# Supplementary material for: Biological effects of dental resin composites on human gingival fibroblasts: a systematic review of in vitro evidence
Source: Clin Oral Investig. 2026 Oct 5;30(10):510. doi: 10.1007/s00784-026-07149-3 (PMC13638730; doi:10.1007/s00784-026-07149-3)
Supplement: Supplementary file 1 [file 784_2026_7149_MOESM1_ESM.zip › Table S.6.1.docx]

**Biological Effects of Dental Resin Composites on Human Gingival Fibroblasts: A Systematic Review of *In Vitro* Evidence**

**Elmira Boloori ^1^** (<https://orcid.org/0000-0002-9048-7988>)

**Dagmar E. Slot ^1^** (<https://orcid.org/0000-0001-7234-0037>)

**Bruno G. Loos ^1^** (https://orcid.org/[0000-0002-8794-552X](https://orcid.org/0000-0002-8794-552X))

**Teun J. de Vries ^1^** (<https://orcid.org/0000-0001-6464-5172>)

**G.A. van der Weijden ^1^** (<https://orcid.org/0000-0002-5075-8384>)

**^1^** Department of Periodontology, Academic Centre for Dentistry Amsterdam (ACTA), University of Amsterdam and Vrije Universiteit Amsterdam, Amsterdam, The Netherlands

**Corresponding author:**

E. Boloori,

Academic Centre for Dentistry Amsterdam (ACTA),

University of Amsterdam and Vrije Universiteit Amsterdam

Department of Periodontology

Gustav Mahlerlaan 3004, 1081 LA Amsterdam, The Netherlands

Phone: +(31)20 5980 179

E-mail: e.boloori@acta.nl

Journal: Clinical Oral Investigations

Scoring sheet for QUIN Tool (quality assessment tool for *in vitro* studies)

| Criteria No. | Criteria | Adequately Speciﬁed (Score=2) | Inadequately Speciﬁed (Score=1) | Not Speciﬁed (Score=0) | Not Applicable |
| --- | --- | --- | --- | --- | --- |
| 1 | Clearly stated aims/objectives |  |  |  |  |
| 2 | Detailed explanation of sample size calculation |  |  |  |  |
| 3 | Detailed explanation of sampling technique |  |  |  |  |
| 4 | Details of comparison group |  |  |  |  |
| 5 | Detailed explanation of methodology |  |  |  |  |
| 6 | Operator details |  |  |  |  |
| 7 | Randomization |  |  |  |  |
| 8 | Method of measurement of outcome |  |  |  |  |
| 9 | Outcome assessor details |  |  |  |  |
| 10 | Blinding |  |  |  |  |
| 11 | Statistical analysis |  |  |  |  |
| 12 | Presentation of results |  |  |  |  |

Table S.6.1: The QUIN risk of bias tool

>70%=low risk of bias

50% to 70%=medium risk of bias,

<50%=high risk of bias
