# Supplementary material for: Biological effects of dental resin composites on human gingival fibroblasts: a systematic review of in vitro evidence
Source: Clin Oral Investig. 2026 Oct 5;30(10):510. doi: 10.1007/s00784-026-07149-3 (PMC13638730; doi:10.1007/s00784-026-07149-3)
Supplement: Supplementary file 1 [file 784_2026_7149_MOESM1_ESM.zip › Table S.6.2.docx]

**Biological Effects of Dental Resin Composites on Human Gingival Fibroblasts: A Systematic Review of *In Vitro* Evidence**

**Elmira Boloori ^1^** (<https://orcid.org/0000-0002-9048-7988>)

**Dagmar E. Slot ^1^** (<https://orcid.org/0000-0001-7234-0037>)

**Bruno G. Loos ^1^** (https://orcid.org/[0000-0002-8794-552X](https://orcid.org/0000-0002-8794-552X))

**Teun J. de Vries ^1^** (<https://orcid.org/0000-0001-6464-5172>)

**G.A. van der Weijden ^1^** (<https://orcid.org/0000-0002-5075-8384>)

**^1^** Department of Periodontology, Academic Centre for Dentistry Amsterdam (ACTA), University of Amsterdam and Vrije Universiteit Amsterdam, Amsterdam, The Netherlands

**Corresponding author:**

E. Boloori,

Academic Centre for Dentistry Amsterdam (ACTA),

University of Amsterdam and Vrije Universiteit Amsterdam

Department of Periodontology

Gustav Mahlerlaan 3004, 1081 LA Amsterdam, The Netherlands

Phone: +(31)20 5980 179

E-mail: e.boloori@acta.nl

Journal: Clinical Oral Investigations

| Author | Criteria no. | | | | | | | | | | | | Sum | |
| --- | --- | --- | --- | --- | --- | --- | --- | --- | --- | --- | --- | --- | --- | --- |
|  | 1 | 2 | 3 | 4 | 5 | 6 | 7 | 8 | 9 | 10 | 11 | 12 |  |  |
| **1. Caughman et al. 1991** | 2 | 0 | 2 | 2 | 2 | 0 | NA | 2 | 0 | NA | 2 | 1 | 13/20  0.65 | 65% |
| **2. Willerhausen et al. 1999** | 2 | 0 | 2 | 2 | 2 | 0 | NA | 2 | 0 | NA | 2 | 2 | 14/20  0.70 | 70% |
| **3. Stanislawski et al. 2000** | 2 | 0 | 2 | 2 | 2 | 0 | NA | 2 | 0 | NA | 2 | 1 | 11/20  0.55 | 55% |
| **4. Huang et al. 2002** | 2 | 0 | 2 | 2 | 2 | 0 | NA | 2 | 0 | NA | 2 | 2 | 14/20  0.7 | 70% |
| **5. Sigusch et al. 2007** | 2 | 0 | 2 | 2 | 2 | 0 | NA | 2 | 0 | NA | 2 | 2 | 14/20  0.70 | 70% |
| **6. Franz et al. 2007** | 2 | 0 | 2 | 2 | 2 | 0 | NA | 2 | 0 | NA | 2 | 2 | 14/20  0.7 | 70% |
| **7. Deb et al. 2011** | 2 | 0 | 2 | 2 | 2 | 0 | NA | 2 | 0 | NA | 2 | 1 | 13/20  0.65 | 65% |
| **8. Sigusch et al. 2012** | 2 | 0 | 2 | 2 | 2 | 0 | NA | 2 | 0 | NA | 2 | 2 | 14/20  0.70 | 70% |
| **9. Tadin et al. 2014** | 2 | 0 | 2 | 2 | 2 | 0 | NA | 2 | 0 | NA | 2 | 2 | 14/20  0.70 | 70% |
| **10. Ajaj et al. 2013** | 2 | 0 | 1 | 2 | 2 | 0 | NA | 2 | 0 | NA | 2 | 2 | 13/20  0.65 | 65% |
| **11. Attik et al. 2014** | 2 | 0 | 2 | 2 | 2 | 0 | NA | 2 | 0 | NA | 2 | 2 | 14/20  0.7 | 70% |
| **12. Schulz et al. 2015** | 2 | 0 | 2 | 2 | 2 | 0 | NA | 2 | 0 | NA | 2 | 2 | 14/20  0.7 | 70% |
| **13. Attik et al. 2017** | 2 | 0 | 2 | 2 | 2 | 0 | NA | 2 | 0 | NA | 2 | 2 | 14/20  0.70 | 70% |
| **14. Koohpeima et al. 2017** | 2 | 0 | 2 | 2 | 2 | 0 | NA | 2 | 0 | NA | 2 | 2 | 14/20  0.7 | 70% |
| **15. Frese et al. 2018** | 2 | 0 | 2 | 2 | 2 | 0 | NA | 2 | 0 | NA | 2 | 2 | 14/20  0.70 | 70% |
| **16. Goncalves et al. 2018** | 2 | 0 | 2 | 2 | 2 | 0 | NA | 2 | 0 | NA | 2 | 2 | 14/20  0.70 | 70% |
| **17. Hussain et al. 2017** | 2 | 0 | 2 | 2 | 2 | 0 | NA | 2 | 0 | NA | 2 | 2 | 14/20  0.70 | 70% |
| **18. Yang et al. 2017** | 2 | 0 | 2 | 2 | 2 | 0 | NA | 2 | 0 | NA | 2 | 2 | 14/20  0.70 | 70% |
| **19. Jerg et al. 2017** | 2 | 0 | 2 | 2 | 2 | 0 | NA | 2 | 0 | NA | 2 | 2 | 14/20  0.70 | 70% |
| **20. Johnsen et al. 2017** | 2 | 0 | 2 | 2 | 2 | 0 | NA | 2 | 0 | NA | 2 | 2 | 14/20  0.70 | 70% |
| **21. Liu et al. 2018** | 2 | 0 | 2 | 2 | 2 | 0 | NA | 2 | 0 | NA | 2 | 2 | 14/20  0.70 | 70% |
| **22. Schubert et al. 2018** | 2 | 0 | 2 | 2 | 2 | 0 | NA | 2 | 0 | NA | 2 | 1 | 13/20  0.65 | 65% |
| **23. Celik et al. 2019** | 2 | 0 | 2 | 2 | 2 | 0 | NA | 2 | 0 | NA | 2 | 2 | 14/20  0.70 | 70% |
| **24. Fujioka-Kobayashi et al. 2019** | 2 | 0 | 2 | 2 | 2 | 2 | NA | 2 | 0 | NA | 2 | 2 | 14/20  0.70 | 70% |
| **25. Kavuncu et al. 2020** | 2 | 0 | 2 | 2 | 0 | 0 | NA | 2 | 0 | NA | 2 | 2 | 14/20  0.70 | 70% |
| **26. Alamoush et al. 2020** | 2 | 0 | 2 | 2 | 1 | 0 | NA | 2 | 0 | NA | 2 | 2 | 14/20  0.70 | 70% |
| **27. Haugen et al. 2020** | 2 | 0 | 2 | 2 | 2 | 0 | NA | 2 | 0 | NA | 2 | 2 | 14/20  0.70 | 70% |
| **28. Ilie et al. 2021** | 2 | 0 | 2 | 2 | 2 | 0 | NA | 2 | 0 | NA | 2 | 2 | 14/20  0.70 | 70% |
| **29. Beltrami et al. 2021** | 2 | 0 | 2 | 2 | 2 | 0 | NA | 2 | 0 | NA | 2 | 2 | 14/20  0.70 | 70% |
| **30. Roussou et al. 2021** | 2 | 0 | 2 | 2 | 2 | 0 | NA | 2 | 0 | NA | 2 | 2 | 14/20  0.70 | 70% |
| **31. De Angelis et al. 2021** | 2 | 0 | 2 | 2 | 2 | 0 | NA | 2 | 0 | NA | 2 | 2 | 14/20  0.7 | 70% |
| **32. Hoseinifar et al. 2022** | 2 | 0 | 2 | 2 | 2 | 0 | NA | 2 | 0 | NA | 2 | 2 | 14/20  0.70 | 70% |
| **33. Aydın et al. 2023** | 2 | 0 | 2 | 2 | 2 | 0 | NA | 2 | 0 | NA | 2 | 2 | 14/20  0.70 | 70% |
| **34. Ghezzi et al. 2024** | 2 | 0 | 2 | 2 | 2 | 2 | NA | 2 | 0 | NA | 2 | 2 | 16/20  0.8 | 80% |
| **35. Flottes , Dursun 2025** | 2 | 0 | 2 | 2 | 2 | 0 | NA | 2 | 0 | NA | 2 | 2 | 14/20  0.7 | 70% |
| **36. Mahmoud 2025** | 2 | 0 | 2 | 2 | 2 | 0 | NA | 2 | 0 | NA | 2 | 2 | 14/20  0.70 | 70% |
| **37. Hashemian, 2025** | 2 | 0 | 1 | 2 | 2 | 0 | NA | 2 | 0 | NA | 2 | 2 | 13/20  0.65 | 65% |
| **38. Erdal, Karadag, 2025** | 2 | 0 | 2 | 2 | 2 | 0 | NA | 2 | 0 | NA | 2 | 2 | 14/20  0.70 | 70% |

Table S.6.2: The assessment of the included studies using QUIN risk of bias tool

A total of 10 items were used to evaluate the risk of bias for each study, as items 7 and 10 were not applicable
