# Supplementary material for: Biological effects of dental resin composites on human gingival fibroblasts: a systematic review of in vitro evidence
Source: Clin Oral Investig. 2026 Oct 5;30(10):510. doi: 10.1007/s00784-026-07149-3 (PMC13638730; doi:10.1007/s00784-026-07149-3)
Supplement: Supplementary file 1 [file 784_2026_7149_MOESM1_ESM.zip › Table S.7.docx]

**Biological Effects of Dental Resin Composites on Human Gingival Fibroblasts: A Systematic Review of *In Vitro* Evidence**

**Elmira Boloori ^1^** (<https://orcid.org/0000-0002-9048-7988>)

**Dagmar E. Slot ^1^** (<https://orcid.org/0000-0001-7234-0037>)

**Bruno G. Loos ^1^** (https://orcid.org/[0000-0002-8794-552X](https://orcid.org/0000-0002-8794-552X))

**Teun J. de Vries ^1^** (<https://orcid.org/0000-0001-6464-5172>)

**G.A. van der Weijden ^1^** (<https://orcid.org/0000-0002-5075-8384>)

**^1^** Department of Periodontology, Academic Centre for Dentistry Amsterdam (ACTA), University of Amsterdam and Vrije Universiteit Amsterdam, Amsterdam, The Netherlands

**Corresponding author:**

E. Boloori,

Academic Centre for Dentistry Amsterdam (ACTA),

University of Amsterdam and Vrije Universiteit Amsterdam

Department of Periodontology

Gustav Mahlerlaan 3004, 1081 LA Amsterdam, The Netherlands

Phone: +(31)20 5980 179

E-mail: e.boloori@acta.nl

Journal: Clinical Oral Investigations

| Author | Volume specimen  (mm^3^ or mg) | Specimen Ø (mm) | Specimen H (mm) |
| --- | --- | --- | --- |
| 1. Caughman et al. 1991 | 78.54 | Ø 10 | 1 |
| 2. Willerhausen et al. 1999 | 0.1-0.12 mg |  |  |
| 3. Stanislawski et al. 2000 | 283.53 | Ø19 | 1 |
| 4. Huang et al. 2002 | 24.50 |  | 3.5x3.5x2 |
| 5. Sigusch et al. 2007 | 25.13 | Ø4 | 2 |
| 6. Franz et al. 2007 | 25.13 | Ø4 | 2 |
| 7. Deb et al. 2011 | 3.14 | Ø2 | 1 |
| 8. Sigusch et al. 2012 | 25.13 | Ø4 | 2 |
| 9. Tadin et al. 2014 | 0.1-0.2 mg |  |  |
| 10. Ajaj et al. 2013 | Unclear |  |  |
| 11. Attik et al. 2014 | Unclear | Ø 2 |  |
| 12. Schulz et al. 2015 | 76.97 | Ø7 | 2 |
| 13. Attik et al. 2017 | Unclear | Ø2 |  |
| 14. Koohpeima et al. 2017 | 39.27 | Ø5 | 2 |
| 15. Frese et al. 2018 | 29.45 | Ø5 | 1.5 |
| 16. Goncalves et al. 2018 | 19.63 | Ø5 | 1 |
| 17. Hussain et al. 2017 | 76.6 mg |  |  |
| 18. Yang et al. 2017 | 157.08 | Ø10 | 2 |
| 19. Jerg et al. 2017 | 56.55 | Ø6 | 2 |
| 20. Johnsen et al. 2017 | 100 |  | 25x2x2 |
| 21. Liu et al. 2018 | 176.71 | Ø15 | 1 |
| 22. Schubert et al. 2018 | 56.55 | Ø6 | 2 |
| 23. Celik et al. 2019 | 157.08 | Ø10 | 2 |
| 24. Fujioka-Kobayashi et al. 2019 | 39.27 | Ø5 | 2 |
| 25. Kavuncu et al. 2020 | 76.97 | Ø7 | 2 |
| 26. Alamoush et al. 2020 | 125 |  | 5x5x5 |
| 27. Haugen et al. 2020 | 502.65 | Ø4 | 10 |
| 28. Ilie et al. 2021 | 39.27 | Ø5 | 2 |
| 29. Beltrami et al. 2021 | 76.97 | Ø7 | 2 |
| 30. Roussou et al. 2021 | 39.27 | Ø5 | 2 |
| 31. De Angelis et al. 2021 | 39.27 | Ø5 | 2 |
| 32. Hoseinifar et al. 2022 | 25.13 | Ø4 | 2 |
| 33. Aydın et al. 2023 | 192 |  | 12x8x2 |
| 34. Ghezzi et al. 2024 | Unclear |  |  |
| 35. Flottes , Dursun 2025 | 28.27 | Ø 3 | 4 |
| 36. Mahmoud 2025 | 39.27 | Ø5 | 2 |
| 37. Hashemian, 2025 | 56.55 | Ø6 | 2 |
| 38. Erdal, Karadag, 2025 | 76.97 | Ø7 | 2 |

Table S.7: Calculation of the volume of samples
