# Supplementary material for: Biological effects of dental resin composites on human gingival fibroblasts: a systematic review of in vitro evidence
Source: Clin Oral Investig. 2026 Oct 5;30(10):510. doi: 10.1007/s00784-026-07149-3 (PMC13638730; doi:10.1007/s00784-026-07149-3)
Supplement: Supplementary file 1 [file 784_2026_7149_MOESM1_ESM.zip › Table S.8.docx]

**Biological Effects of Dental Resin Composites on Human Gingival Fibroblasts: A Systematic Review of *In Vitro* Evidence**

**Elmira Boloori ^1^** (<https://orcid.org/0000-0002-9048-7988>)

**Dagmar E. Slot ^1^** (<https://orcid.org/0000-0001-7234-0037>)

**Bruno G. Loos ^1^** (https://orcid.org/[0000-0002-8794-552X](https://orcid.org/0000-0002-8794-552X))

**Teun J. de Vries ^1^** (<https://orcid.org/0000-0001-6464-5172>)

**G.A. van der Weijden ^1^** (<https://orcid.org/0000-0002-5075-8384>)

**^1^** Department of Periodontology, Academic Centre for Dentistry Amsterdam (ACTA), University of Amsterdam and Vrije Universiteit Amsterdam, Amsterdam, The Netherlands

**Corresponding author:**

E. Boloori,

Academic Centre for Dentistry Amsterdam (ACTA),

University of Amsterdam and Vrije Universiteit Amsterdam

Department of Periodontology

Gustav Mahlerlaan 3004, 1081 LA Amsterdam, The Netherlands

Phone: +(31)20 5980 179

E-mail: e.boloori@acta.nl

Journal: Clinical Oral Investigations

| Authors | Resin composite | Cytotoxicity (necrosis/apoptosis) | | Viability | | | | | | Markers | | | | | | Cell morphology | Attachment | | Proliferation  /Adhesion | Genotoxicity/ modification | ROS |
| --- | --- | --- | --- | --- | --- | --- | --- | --- | --- | --- | --- | --- | --- | --- | --- | --- | --- | --- | --- | --- | --- |
| **Ajaj et al.**  **2013** | 3D-direct | NA | | 50ul | | | 0 | | | NA | | | | | | NA | NA | | NA | NA | NA |
|  |  |  |  | 100ul | | | 0 | | |  |  |  |  |  |  |  |  |  |  |  |  |
| **Ajaj et al.**  **2013** | 4 Seasons | NA | | 50ul | | | 0 | | | NA | | | | | | NA | NA | | NA | NA | NA |
|  |  |  |  | 100ul | | | 0 | | |  |  |  |  |  |  |  |  |  |  |  |  |
| **Johnsen et al.**  **2017** | 4U | 24h | 48h | NA | | | | | | NA | | | | | | 0 | 0 | | NA | NA | NA |
|  |  | 0 | + |  |  |  |  |  |  |  |  |  |  |  |  |  |  |  |  |  |  |
| **Frese et al.**  **2018** | Admira /fusion | NA | | NA | | | | | | NA | | | | | | NA | - | | NA | NA | NA |
| **Yang et al.**  **2017** |  | NA | | 0 | | | | | | NA | | | | | | NA | NA | | NA | 0 | NA |
| **Schubert et al.**  **2018** |  | NA | | 48h | | 72h | | | | NA | | | | | | NA | NA | | NA | NA | NA |
|  |  |  |  | - | | - | | | |  |  |  |  |  |  |  |  |  |  |  |  |
| **Kavuncu et al.**  **2020** |  | 24h | 1w | 24h | | 1w | | | | NA | | | | | | NA | NA | | NA | NA | NA |
|  |  | 0 | 0 | 0 | | 0 | | | |  |  |  |  |  |  |  |  |  |  |  |  |
| **Beltrami et al. 2021** |  | NA | | 48h | | 72h | | | | NA | | | | | | NA | NA | | NA | NA | NA |
|  |  |  |  | - | | - | | | |  |  |  |  |  |  |  |  |  |  |  |  |
| **Hoseinifar et al. 2022** |  | 0 | | NA | | | | | | NA | | | | | | NA | NA | | NA | NA | NA |
| **Yang et al.**  **2017** | AP-X | NA | | 0 | | | | | | NA | | | | | | NA | NA | | NA | 0 | NA |
| **Liu et al.**  **2018** |  | NA | | 0 | | | | | | NA | | | | | | NA | NA | | NA | NA | + |
| **Schulz et al.**  **2015** | Ceram X | 24h | 1w | NA | | | | | |  | 1d | | | 7d | | NA | 24h | 1w | NA | NA | NA |
|  |  | 0 | 0 |  |  |  |  |  |  | FLG | 0 | | | 0 | |  | 0 | 0 |  |  |  |
|  |  |  |  |  |  |  |  |  |  | IVL | 0 | | | 0 | |  |  |  |  |  |  |
|  |  |  |  |  |  |  |  |  |  | CASP3 | 0 | | | 0 | |  |  |  |  |  |  |
|  |  |  |  |  |  |  |  |  |  | CASP 8 | 0 | | | 0 | |  |  |  |  |  |  |
|  |  |  |  |  |  |  |  |  |  | CASP 9 | 0 | | | 0 | |  |  |  |  |  |  |
|  |  |  |  |  |  |  |  |  |  | IL 6 | 0 | | | 0 | |  |  |  |  |  |  |
|  |  |  |  |  |  |  |  |  |  | IL 18 | - | | | 0 | |  |  |  |  |  |  |
|  |  |  |  |  |  |  |  |  |  | ANX AS | 0 | | | 0 | |  |  |  |  |  |  |
|  |  |  |  |  |  |  |  |  |  | ITGB3 | 0 | | | 0 | |  |  |  |  |  |  |
|  |  |  |  |  |  |  |  |  |  | MKi67 | 0 | | | 0 | |  |  |  |  |  |  |
| **Jerg et al.**  **2017** |  | NA | | NA | | | | | |  | 1d | | | 3d | | 0 | 0 | |  | NA | NA |
|  |  |  |  |  |  |  |  |  |  | VIM | 0 | | | 0 | |  |  | |  |  |  |
|  |  |  |  |  |  |  |  |  |  | LOX | 0 | | | 0 | |  |  | |  |  |  |
|  |  |  |  |  |  |  |  |  |  |  | 0 | | | 0 | |  |  | |  |  |  |
|  |  |  |  |  |  |  |  |  |  | ITGB1 | 0 | | | 0 | |  |  | |  |  |  |
|  |  |  |  |  |  |  |  |  |  | pERK1/2 | 0 | | | 0 | |  |  | |  |  |  |
|  |  |  |  |  |  |  |  |  |  | MKi67 | 0 | | | - | |  |  | | - |  |  |
|  |  |  |  |  |  |  |  |  |  | MMP1 | 0 | | | 0 | |  |  | |  |  |  |
|  |  |  |  |  |  |  |  |  |  | IL 6 | 0 | | | 0 | |  |  | |  |  |  |
| **Willerhausen et al.**  **1999** | Charisma / Topaz | NA | | NA | | | | | | PGE­_2_ | | | 0 | | | NA | NA | | NA | NA | NA |
| **Kavuncu et al.**  **2020** |  | 24h | 1w | 24h | | 1w | | | | NA | | | | | | NA | NA | | NA | NA | NA |
|  |  | + | - | - | | - | | | |  |  |  |  |  |  |  |  |  |  |  |  |
| **Erdal and Karadag, 2025** | Charisma Diamond One | NA | | 1d | | 7d | | | | NA | | | | | | NA | NA | | NA | NA | + |
|  |  |  |  | - | | - | | | |  |  |  |  |  |  |  |  |  |  |  |  |
| **De Angelis et al. 2021** | Clearfil Majesty ES-2 | NA | | 24h | | 14d | | | | NA | | | | | | NA | NA | | NA | 0 | 0 |
|  |  |  |  | 0 | | 0 | | | |  |  |  |  |  |  |  |  |  |  |  |  |
| **Erdal and Karadag, 2025** |  | NA | | 1d | | 7d | | | | NA | | | | | | NA | NA | | NA | NA | 0 |
|  |  |  |  | 0 | | - | | | |  |  |  |  |  |  |  |  |  |  |  |  |
| **Aydin et al. 2023** | Clearfil Majesty posterior | 0 | | 0 | | | | | |  | | | | | |  |  | |  | 0 |  |
| **Sigusch et al.**  **2007** | Durafill VS | NA | | 1d | | 5d | | | | NA | | | | | | NA | NA | | NA | NA | NA |
|  |  |  |  | 0 | | 0 | | | |  |  |  |  |  |  |  |  |  |  |  |  |
| **Ajaj et al.**  **2013** |  | NA | | 50ul | | 0 | | | | NA | | | | | | NA | NA | | NA | NA | NA |
|  |  |  |  | 100ul | | 0 | | | |  |  |  |  |  |  |  |  |  |  |  |  |
| **Attic et al.**  **2014** | ELS | NA | | - | | | | | | NA | | | | | | 0 | NA | | NA | NA | NA |
| **Attik et al.**  **2016** |  | 0 | | 0 | | | | | | NA | | | | | | NA | NA | | NA | NA | NA |
| **Roussou et al. 2021** | ENA Hri | NA | | 24 | | 72 | | | | NA | | | | | | NA | NA | | NA | NA | NA |
|  |  |  |  | 0 | | - | | | |  |  |  |  |  |  |  |  |  |  |  |  |
| **De Angelis et al. 2021** |  | NA | | 24h | | 14d | | | | NA | | | | | | NA | NA | | NA | + | + |
|  |  |  |  | - | | - | | | |  |  |  |  |  |  |  |  |  |  |  |  |
| **Beltrami et al. 2021** | Enamel Plus HRi | NA | | 48h | | 72h | | | | NA | | | | | | NA | NA | | NA | NA | NA |
|  |  |  |  | - | | - | | | |  |  |  |  |  |  |  |  |  |  |  |  |
| **De Angelis et al. 2021** | Enamel BioFunction | NA | | 24h | | 14d | | | | NA | | | | | | NA | NA | | NA | 0 | 0 |
|  |  |  |  |  | | 0 | | | |  |  |  |  |  |  |  |  |  |  |  |  |
| **Beltrami et al. 2021** | Enamel Plus HRi  Bio Function Enamel | NA | | 48h | | 72h | | | | NA | | | | | | NA | NA | | NA | NA | NA |
|  |  |  |  | - | | - | | | |  |  |  |  |  |  |  |  |  |  |  |  |
| **Erdal and Karadag, 2025** | Essentia Universal | NA | | 1 d | | 7 d | | | | NA | | | | | | NA | NA | | NA | NA | 0 |
|  |  |  |  | 0 | | - | | | |  |  |  |  |  |  |  |  |  |  |  |  |
| **Kavuncu et al.**  **2020** | EsteliteQuickSigma | 24h | 1w | 24h | | 1w | | | | NA | | | | | | NA | NA | | NA | NA | NA |
|  |  | 0 | 0 | 0 | | 0 | | | |  |  |  |  |  |  |  |  |  |  |  |  |
| **Ajaj et al.**  **2013** | Esthet X | NA | | 50ul | | | 0 | | | NA | | | | | | NA | NA | | NA | NA | NA |
|  |  |  |  | 100ul | | | 0 | | |  |  |  |  |  |  |  |  |  |  |  |  |
| **Yang et al.**  **2017** |  | NA | | 0 | | | | | | NA | | | | | | NA | NA | | NA | + | NA |
| **Goncalves et al.**  **2018** | Filtec Z350 /Flowable | NA | | - | | | | | | NA | | | | | | NA | NA | | NA | NA | NA |
| **Liu et al.**  **2018** |  | NA | | 0 | | | | | | NA | | | | | | NA | NA | | NA | NA | + |
| **Mahmoud et al. 2025** |  | NA | | - | | | | | | IL-6 | | + | | | | NA | NA | | NA | + | NA |
|  |  |  |  |  |  |  |  |  |  | IL-1B | | + | | | |  |  |  |  |  |  |
| **Hashemian et al. 2025** |  | NA | | Depending on the distance  - (>2 mm) | | | | | | NA | | | | | | NA | NA | | NA | NA | NA |
| **Liu et al.**  **2018** | Filtek P60 | NA | | 0 | | | | | | NA | | | | | | NA | NA | | NA | NA | + |
| **Deb et al.**  **2011** |  | NA | | 0 | | | | | | NA | | | | | | NA | NA | | NA | NA | NA |
| **Schulz et al.**  **2015** | Filtek Silorane | 24h | 1w | NA | | | | | |  | 1d | | | 7d | | NA | 24h | 1w | NA | NA | NA |
|  |  | 0 | 0 |  |  |  |  |  |  | FLG | 0 | | | 0 | |  | 0 | 0 |  |  |  |
|  |  |  |  |  |  |  |  |  |  | IVL | 0 | | | 0 | |  |  |  |  |  |  |
|  |  |  |  |  |  |  |  |  |  | CASP3 | 0 | | | 0 | |  |  |  |  |  |  |
|  |  |  |  |  |  |  |  |  |  | CASP 8 | 0 | | | 0 | |  |  |  |  |  |  |
|  |  |  |  |  |  |  |  |  |  | CASP 9 | 0 | | | 0 | |  |  |  |  |  |  |
|  |  |  |  |  |  |  |  |  |  | IL 6 | 0 | | | + | |  |  |  |  |  |  |
|  |  |  |  |  |  |  |  |  |  | IL 18 | - | | | 0 | |  |  |  |  |  |  |
|  |  |  |  |  |  |  |  |  |  | ANX AS | 0 | | | 0 | |  |  |  |  |  |  |
|  |  |  |  |  |  |  |  |  |  | ITGB3 | 0 | | | 0 | |  |  |  |  |  |  |
|  |  |  |  |  |  |  |  |  |  | MKi67 | 0 | | | 0 | |  |  |  |  |  |  |
| **Jerg et al.**  **2017** |  | NA | | NA | | | | | |  | 1d | | | 3d | | 0 | 0 | |  | NA | NA |
|  |  |  |  |  |  |  |  |  |  | VIM | 0 | | | 0 | |  |  | |  |  |  |
|  |  |  |  |  |  |  |  |  |  | LOX | 0 | | | + | |  |  | |  |  |  |
|  |  |  |  |  |  |  |  |  |  | ITGB1 | 0 | | | - | |  |  | |  |  |  |
|  |  |  |  |  |  |  |  |  |  | pERK1/2 | 0 | | | 0 | |  |  | |  |  |  |
|  |  |  |  |  |  |  |  |  |  | MKi67 | 0 | | | - | |  |  | | - |  |  |
|  |  |  |  |  |  |  |  |  |  | MMP1 | 0 | | | 0 | |  |  | |  |  |  |
|  |  |  |  |  |  |  |  |  |  | IL 6 | 0 | | | 0 | |  |  | |  |  |  |
| **Ajaj et al.**  **2013** | Filtek Supreme XTE/plus | NA | | 50ul | | | 0 | | | NA | | | | | | NA | NA | | NA | NA | NA |
|  |  |  |  | 100ul | | | 0 | | |  |  |  |  |  |  |  |  |  |  |  |  |
| **Schulz et al.**  **2015** |  | 24h | 1w | NA | | | | | |  | 1d | | | 7d | | NA | 24h | 1w | NA | NA | NA |
|  |  | 0 | 0 |  |  |  |  |  |  | FLG | 0 | | | 0 | |  | 0 | 0 |  |  |  |
|  |  |  |  |  |  |  |  |  |  | IVL | 0 | | | 0 | |  |  |  |  |  |  |
|  |  |  |  |  |  |  |  |  |  | CASP3 | 0 | | | 0 | |  |  |  |  |  |  |
|  |  |  |  |  |  |  |  |  |  | CASP 8 | 0 | | | 0 | |  |  |  |  |  |  |
|  |  |  |  |  |  |  |  |  |  | CASP 9 | 0 | | | 0 | |  |  |  |  |  |  |
|  |  |  |  |  |  |  |  |  |  | IL 6 | 0 | | | 0 | |  |  |  |  |  |  |
|  |  |  |  |  |  |  |  |  |  | IL 18 | - | | | 0 | |  |  |  |  |  |  |
|  |  |  |  |  |  |  |  |  |  | ANX AS | 0 | | | 0 | |  |  |  |  |  |  |
|  |  |  |  |  |  |  |  |  |  | ITGB3 | 0 | | | 0 | |  |  |  |  |  |  |
|  |  |  |  |  |  |  |  |  |  | MKi67 | 0 | | | 0 | |  |  |  |  |  |  |
| **Frese et al.**  **2017** |  | NA | | NA | | | | | | NA | | | | | | NA | - | | NA | NA | NA |
| **Jerg et al.**  **2017** |  | NA | | NA | | | | | |  | 1d | | | 3d | | 0 | 0 | |  | NA | NA |
|  |  |  |  |  |  |  |  |  |  | VIM | 0 | | | 0 | |  |  | |  |  |  |
|  |  |  |  |  |  |  |  |  |  | LOX | 0 | | | + | |  |  | |  |  |  |
|  |  |  |  |  |  |  |  |  |  | ITGB1 | 0 | | | 0 | |  |  | |  |  |  |
|  |  |  |  |  |  |  |  |  |  | pERK1/2 | 0 | | | - | |  |  | | - |  |  |
|  |  |  |  |  |  |  |  |  |  | MKi67 | 0 | | | 0 | |  |  | |  |  |  |
|  |  |  |  |  |  |  |  |  |  | MMP1 | 0 | | | 0 | |  |  | |  |  |  |
|  |  |  |  |  |  |  |  |  |  | IL 6 | 0 | | | 0 | |  |  | |  |  |  |
| **Schubert et al.**  **2018** |  | NA | | 48h | | 72h | | | | NA | | | | | | NA | NA | | NA | NA | NA |
|  |  |  |  | - | | - | | | |  |  |  |  |  |  |  |  |  |  |  |  |
| **Sigusch et al.**  **2006** | Filtek Z250 | NA | | 1d | | 5d | | | | NA | | | | | | NA | NA | | NA | NA | NA |
|  |  |  |  | 0 | | 0 | | | |  |  |  |  |  |  |  |  |  |  |  |  |
| **Sigusch et al.**  **2012** |  | NA | | 0 | | | | | | NA | | | | | | NA | NA | | NA | NA | NA |
| **Attik et al.**  **2016** |  | 0 | | 0 | | | | | | NA | | | | | | NA | NA | | NA | NA | NA |
| **Koohpeima et al.**  **2017** |  | NA | | - | | | | | | NA | | | | | | NA | NA | | NA | NA | NA |
| **Hussain et al.**  **2017** |  | 24h | 48h | NA | | | | | | NA | | | | | | 0 | NA | | NA | NA | NA |
|  |  | 0 | 0 |  |  |  |  |  |  |  |  |  |  |  |  |  |  |  |  |  |  |
| **Johnsen et al.**  **2017** |  | 24h | 48h | NA | | | | | | NA | | | | | | 0 | 0 | | NA | NA | NA |
|  |  | 0 | 0 |  |  |  |  |  |  |  |  |  |  |  |  |  |  |  |  |  |  |
| **Celik et al.**  **2019** |  | NA | | 0d | 7d | | | 21d | | NA | | | | | | NA | NA | | NA | NA | 0 |
|  |  |  |  | - | - | | | - | |  |  |  |  |  |  |  |  |  |  |  |  |
| **Attik et al.**  **2016** | G-aenial anterior/posterior | 0 | | 0 | | | | | | NA | | | | | | NA | NA | | NA | NA | NA |
| **Beltrami et al. 2021** |  | NA | | 48h | | 72h | | | | NA | | | | | | NA | NA | | NA | NA | NA |
|  |  |  |  | - | | - | | | |  |  |  |  |  |  |  |  |  |  |  |  |
| **Ghezzi et al. 2024** | anterior | 0 | | 0 | | | | | | IL-6 | | 0 | | | | NA | NA | | NA | NA | NA |
|  |  |  |  |  |  |  |  |  |  | TGF-B | | - | | | |  |  |  |  |  |  |
| **Sigusch et al.**  **2006** | GrandiO /SO | NA | | 1d | | 5d | | | | NA | | | | | | NA | NA | | NA | NA | NA |
|  |  |  |  | 0 | | 0 | | | |  |  |  |  |  |  |  |  |  |  |  |  |
| **Sigusch et al.**  **2011** |  | NA | | 0 | | | | | | NA | | | | | | NA | NA | | NA | NA | NA |
| **Ajaj et al.**  **2013** |  | NA | | 50ul | | | 0 | | | NA | | | | | | NA | NA | | NA | NA | NA |
|  |  |  |  | 100ul | | | 0 | | |  |  |  |  |  |  |  |  |  |  |  |  |
| **Schubert et al.**  **2018** |  | NA | | 48h | | 72h | | | | NA | | | | | | NA | NA | | NA | NA | NA |
|  |  |  |  | - | | - | | | |  |  |  |  |  |  |  |  |  |  |  |  |
| **Alamoush et a.**  **2020** |  | 0 | | 5d –  10d – | | | | | | NA | | | | | | NA | NA | | NA | NA | NA |
| **De Angelis** |  | NA | | 24h | | 14d | | | | NA | | | | | | NA | NA | | NA | 0 | 0 |
|  |  |  |  | 0 | | 0 | | | |  |  |  |  |  |  |  |  |  |  |  |  |
| **Hoseinifar et al. 2022** |  | + | | NA | | | | | | NA | | | | | | NA | NA | | NA | NA | NA |
| **Ajaj et al.**  **2013** | Heliomolar | NA | | 50ul | | | 0 | | | NA | | | | | | NA | NA | | NA | NA | NA |
|  |  |  |  | 100ul | | | 0 | | |  |  |  |  |  |  |  |  |  |  |  |  |
| **Deb et al.**  **2011** |  | NA | | 0 | | | | | | NA | | | | | | NA | NA | | NA | NA | NA |
| **Franz et al.**  **2007** | Herculite XRV Enamel | + | | NA | | | | | | NA | | | | | | NA | NA | | NA | NA | NA |
| **Deb et al.**  **2011** |  | NA | | 0 | | | | | | NA | | | | | | NA | NA | | NA | NA | NA |
| **Ajaj et al.**  **2013** | ICE | NA | | 50ul | | | 0 | | | NA | | | | | | NA | NA | | NA | NA | NA |
|  |  |  |  | 100ul | | | 0 | | |  |  |  |  |  |  |  |  |  |  |  |  |
| **Tadin et al.**  **2013** | Kalore | 0 | | NA | | | | | | NA | | | | | | NA | NA | | NA | + | NA |
| **Caughman et al.**  **1991** | Multifil | 0 ? | | NA | | | | | | NA | | | | | | NA | NA | | NA | NA | NA |
| **Caughman et al.**  **1991** | P50 | 0 ? | | NA | | | | | | NA | | | | | | NA | NA | | NA | NA | NA |
| **Caughman et al.**  **1991** | Prisma-Micro-fine | 0 ? | | NA | | | | | | NA | | | | | | NA | NA | | NA | NA | NA |
| **Ajaj et al.**  **2013** | Prisma AP.H | NA | | 50ul | | | 0 | | | NA | | | | | | NA | NA | | NA | NA | NA |
|  |  |  |  | 100ul | | | 0 | | |  |  |  |  |  |  |  |  |  |  |  |  |
| **Ajaj et al.**  **2013** | Quixx | NA | | 50ul | | | 0 | | | NA | | | | | | NA | NA | | NA | NA | NA |
|  |  |  |  | 100ul | | | 0 | | |  |  |  |  |  |  |  |  |  |  |  |  |
| **Yang et al.**  **2017** | QuixFil | NA | | 0 | | | | | | NA | | | | | | NA | NA | | NA | 0 | NA |
| **Ajaj et al.**  **2013** | Rok | NA | | 50ul | | | 0 | | | NA | | | | | | NA | NA | | NA | NA | NA |
|  |  |  |  | 100ul | | | 0 | | |  |  |  |  |  |  |  |  |  |  |  |  |
| **Willerhausen et al.**  **1999** | Solitaire / 2 | NA | | NA | | | | | | PGE­_2_ | | | 0 | | | NA | NA | | NA | NA | NA |
| **Sigusch et al.**  **2006** |  | NA | | 1d | | 5d | | | | NA | | | | | | NA | NA | | NA | NA | NA |
|  |  |  |  | 0 | | 0 | | | |  |  |  |  |  |  |  |  |  |  |  |  |
| **Sigusch et al.**  **2011** |  | NA | | 0 | | | | | | NA | | | | | | NA | NA | | NA | NA | NA |
| **Huang et al.**  **2002** | Spectrum TPH | - | | - | | | | | | NA | | | | | | NA | - | | NA | NA | NA |
| **Deb et al.**  **2011** |  | NA | | 0 | | | | | | NA | | | | | | NA | NA | | NA | NA | NA |
| **Ajaj et al.**  **2013** | SureFil | NA | | 50ul | | | 0 | | | NA | | | | | | NA | NA | | NA | NA | NA |
|  |  |  |  | 100ul | | | 0 | | |  |  |  |  |  |  |  |  |  |  |  |  |
| **Willerhausen et al.**  **1999** | Tetric-Flow /Evo ceram/ ceram | NA | | NA | | | | | | PGE­_2_ | | | 0 | | | NA | NA | | NA | NA | NA |
| **Stanislawski et al.**  **2000** |  | NA | | 0 | | | | | | NA | | | | | | NA | NA | | NA | NA | NA |
| **Ajaj et al.**  **2013** |  | NA | | 50ul | | | 0 | | | NA | | | | | | NA | NA | | NA | NA | NA |
|  |  |  |  | 100ul | | | 0 | | |  |  |  |  |  |  |  |  |  |  |  |  |
| **Attik et al.**  **2016** |  | 0 | | 0 | | | | | | NA | | | | | | NA | NA | | NA | NA | NA |
| **Frese et al.**  **2017** |  | NA | | NA | | | | | | NA | | | | | | 0 | - | | NA | NA | NA |
| **Hussain et al.**  **2017** |  | 24h | 48h | NA | | | | | | NA | | | | | | 0 | NA | | NA | NA | NA |
|  |  | 0 | + |  |  |  |  |  |  |  |  |  |  |  |  |  |  |  |  |  |  |
| **Haugen et al.**  **2020** |  | + | | NA | | | | | | NA | | | | | | 0 | NA | | NA | NA | NA |
| **Flottes, Dursun 2025** |  | NA | | NA | | | | | | Actin, cytoplasmic 1 (ACTB) | | | | | + | NA | NA | | NA | NA | NA |
|  |  |  |  |  |  |  |  |  |  | Actin, cytoplasmic 2 (ACTG1) | | | | | + |  |  |  |  |  |  |
|  |  |  |  |  |  |  |  |  |  | F-actin-capping protein subunit alpha-2 (CAPZA2) | | | | | + |  |  |  |  |  |  |
|  |  |  |  |  |  |  |  |  |  | Endoplasmic reticulum chaperone BiP (HSPA5) | | | | | + |  |  |  |  |  |  |
|  |  |  |  |  |  |  |  |  |  | Endoplasmin (HSP90B1) | | | | | + |  |  |  |  |  |  |
|  |  |  |  |  |  |  |  |  |  | Peroxiredoxin-6 (PRDX6) | | | | | - |  |  |  |  |  |  |
|  |  |  |  |  |  |  |  |  |  | Zyxin (ZYX) | | | | | - |  |  |  |  |  |  |
|  |  |  |  |  |  |  |  |  |  | Pyruvate kinase (PKM) | | | | | - |  |  |  |  |  |  |
|  |  |  |  |  |  |  |  |  |  | Y-box-binding protein 1 (YBX1) | | | | | - |  |  |  |  |  |  |
|  |  |  |  |  |  |  |  |  |  | Collagen alpha-1 (I) chain (COLIA1) | | | | | - |  |  |  |  |  |  |
| **Johnsen et al.**  **2017** | TopDent | 24H 0  48H 0 | | NA | | | | | | NA | | | | | | 0 | NA | | NA | NA | NA |
| **Ajaj et al.**  **2013** | Venus | NA | | 50ul | | | 0 | | | NA | | | | | | NA | NA | | NA | NA | NA |
|  |  |  |  | 100ul | | | 0 | | |  |  |  |  |  |  |  |  |  |  |  |  |
| **Yang et al.**  **2017** |  | NA | | 0 | | | | | | NA | | | | | | NA | NA | | NA | + | NA |
| **Fujioka-Kobayashi et al.**  **2019** |  | NA | | NA | | | | | | IL1 | | | + | | | - | - | | NA | NA | NA |
|  |  |  |  |  |  |  |  |  |  | IL6 | | | + | | |  |  | |  |  |  |
|  |  |  |  |  |  |  |  |  |  | IL8 | | | + | | |  |  | |  |  |  |
|  |  |  |  |  |  |  |  |  |  | IL11 | | | + | | |  |  | |  |  |  |
|  |  |  |  |  |  |  |  |  |  | TGFβ | | | + | | |  |  | |  |  |  |
|  |  |  |  |  |  |  |  |  |  | TNFα | | | 0 | | |  |  | |  |  |  |
|  |  |  |  |  |  |  |  |  |  | Ki67 | | | - | | |  |  | |  |  |  |
|  |  |  |  |  |  |  |  |  |  | Integrin β3 | | | + | | |  |  | |  |  |  |
|  |  |  |  |  |  |  |  |  |  | Caspase 3 | | | 0 | | |  |  | |  |  |  |
|  |  |  |  |  |  |  |  |  |  | Caspase 8 | | | 0 | | |  |  | |  |  |  |
|  |  |  |  |  |  |  |  |  |  | Caspase 9 | | | + | | |  |  | |  |  |  |
|  |  |  |  |  |  |  |  |  |  | Annexin A5 | | | + | | |  |  | |  |  |  |
|  |  |  |  |  |  |  |  |  |  | ADM | | | + | | |  |  | |  |  |  |
|  |  |  |  |  |  |  |  |  |  | PDGF | | | 0 | | |  |  | |  |  |  |
| **Ilie 2021** |  | NA | | 24h | 48h | | | | 72h | NA | | |  | | | NA | NA | | NA | NA | NA |
|  |  |  |  | - | - | | | | 0 |  |  |  |  |  |  |  |  |  |  |  |  |
| **Fujioka-Kobayashi et al.**  **2019** | Venus pearl | NA | | NA | | | | | | IL1 | | | 0 | | | - | - | | NA | NA | NA |
|  |  |  |  |  |  |  |  |  |  | IL6 | | | + | | |  |  | |  |  |  |
|  |  |  |  |  |  |  |  |  |  | IL8 | | | 0 | | |  |  | |  |  |  |
|  |  |  |  |  |  |  |  |  |  | IL11 | | | 0 | | |  |  | |  |  |  |
|  |  |  |  |  |  |  |  |  |  | TGFβ | | | 0 | | |  |  | |  |  |  |
|  |  |  |  |  |  |  |  |  |  | Ki67 | | | 0 | | |  |  | |  |  |  |
|  |  |  |  |  |  |  |  |  |  | Integrin β3 | | | 0 | | |  |  | |  |  |  |
|  |  |  |  |  |  |  |  |  |  | Caspase 3 | | | 0 | | |  |  | |  |  |  |
|  |  |  |  |  |  |  |  |  |  | Caspase 8 | | | 0 | | |  |  | |  |  |  |
|  |  |  |  |  |  |  |  |  |  | Caspase 9 | | | 0 | | |  |  | |  |  |  |
|  |  |  |  |  |  |  |  |  |  | Annexin A5 | | | 0 | | |  |  | |  |  |  |
|  |  |  |  |  |  |  |  |  |  | ADM | | | 0 | | |  |  | |  |  |  |
|  |  |  |  |  |  |  |  |  |  | PDGF | | | 0 | | |  |  | |  |  |  |
| **Ilie et al. 2021** |  | NA | | 24h | 48h | | | | 72h | 48 | | | | | | NA | NA | | NA | NA | NA |
|  |  |  |  | - | 0 | | | | 0 |  |  |  |  |  |  |  |  |  |  |  |  |
| **De Angelis et al. 2021** | Venus Diamond | NA | | 24h | | 14d | | | | NA | | | | | | NA | NA | | NA | + | + |
|  |  |  |  | - | | - | | | |  |  |  |  |  |  |  |  |  |  |  |  |
| **Liu et al.**  **2018** | Vita Zeta + LC | NA | | - | | | | | | - | | | | | | 0 | NA | | NA | NA | + |
| **Stanislawski et al.**  **2000** | Z100MP | NA | | 0 | | | | | | NA | | | | | | NA | NA | | NA | NA | NA |
| **Beltrami et al. 2021** | Omnichroma Blocker | NA | | 48h | | 72h | | | | NA | | | | | | NA | NA | | NA | NA | NA |
|  |  |  |  | - | | - | | | |  |  |  |  |  |  |  |  |  |  |  |  |
| **Beltrami et al. 2021** | Omnichroma | NA | | 48h | | 72h | | | | NA | | | | | | NA | NA | | NA | NA | NA |
|  |  |  |  | - | | - | | | |  |  |  |  |  |  |  |  |  |  |  |  |
| **Mahmoud et al. 2025** |  | NA | | - | | | | | | IL-6 | | + | | | | NA | NA | | NA | + | NA |
|  |  |  |  |  |  |  |  |  |  | IL-1B | | + | | | |  |  |  |  |  |  |
| **Erdal and Karadag, 2025** |  | NA | | 1d | | 7d | | | | NA | | | | | | NA | NA | | NA | NA | - ! |
|  |  |  |  | - | | - | | | |  |  |  |  |  |  |  |  |  |  |  |  |
| **Beltrami et al. 2021** | Enamel Plus HRi | NA | | 48h | | 72h | | | | NA | | | | | | NA | NA | | NA | NA | NA |
|  |  |  |  | - | | - | | | |  |  |  |  |  |  |  |  |  |  |  |  |
| **Erdal and Karadag, 2025** | Vittra APS Unique | NA | | 1 d | | 7d | | | | NA | | | | | | NA | NA | | NA | NA | 0 |
|  |  |  |  | 0 | | - | | | |  |  |  |  |  |  |  |  |  |  |  |  |
| **Erdal and Karadag, 2025** | Zenchroma | NA | | 1d | | 7d | | | | NA | | | | | | NA | NA | | NA | NA | 0 |
|  |  |  |  | 0 | | - | | | |  |  |  |  |  |  |  |  |  |  |  |  |
| **Erdal and Karadag, 2025** | X-tra fil | NA | | 1d | | 7d | | | | NA | | | | | | NA | NA | | NA | NA | 0 |
|  |  |  |  | - | | - | | | |  |  |  |  |  |  |  |  |  |  |  |  |

Table S.8: Extensive table which is reduced to table 1 in the main text

+	significant increased effect compared to control

-	significant decreased effect compared to control

0	no difference

NA	not applicable

?	uncertainty of statistic difference (not mentioned in the article)

Total brands: 52
